# *The stack overflow recommendations dataset (SORD)* – A large-scale curated dataset of recommendations related stack overflow questions, answers and comments

**DOI:** 10.1016/j.dib.2026.112818

**Published:** 2026-05-06

**Authors:** Arjumand Fatima, Onaiza Maqbool

**Affiliations:** Department of Computer Science, Quaid-i-Azam University, Islamabad, Pakistan

**Keywords:** Q&A websites, Software recommendations, Stack exchange, Recommendation systems for software engineering, Crowdsourced knowledge sharing, Software information sites, Community question answering sites, Human factors

## Abstract

Developer discussions particularly on programming related questions answering (Q&A) sites, contain useful information, which, if mined and analysed carefully can be transformed into insightful recommendations for developers about which software to use or prefer over others, matching with one’s requirements for different software development activities. However, there is a long way to go before such a system can be realized. As a first step in this direction, we empirically explored the developers’ discussions on Stack Overflow, one of the most popular Q&A sites among developers, with a particular emphasis on mining software recommendations related insights. We considered the Stack Overflow data dump published in October 2025 containing complete data of the site since 2008. The data extraction process started with the conversion of the gigantic XML files to SQL Server database table records. We then applied a keywords-based filtering approach to identify potential recommendation related queries in questions, and recommendations in answers and comments related to any aspect of software development. The set of keywords comprised of 19 keywords including the term ‘recommend’ along with its 14 synonyms and 4 antonyms. The extracted dataset contains 73.9k (0.31%) questions containing such terms in the question title, 1.1 M (4.69%) questions containing them in the question body, 2.2 M (6.18%) answers and 1.9 M (2.08%) comments because of exact keyword matching. The results further increase in case of substring matching. When enriched with additional metadata e.g. Users, *Badges, Votes* and *Tags* (which are available in the Stack Overflow data dump), the raw dataset presented in this paper can become highly useful for the empirical software engineering and machine learning research community for training models and developing recommendation systems for software engineering. The extracted answers and comments can be mined to extract implicit developer preferences from which ratings can be inferred whereas the extracted recommendation related questions with accepted answers can be transformed into a benchmark for retrieval evaluation of software recommendation systems for developers. The dataset is hosted on Figshare using DOI 10.6084/m9.figshare.30948506 and can be accessed via https://doi.org/10.6084/m9.figshare.30948506 or the GitHub repository.

Specifications Table

**Table:** 

Subject	Computer Sciences
Specific subject area	Empirical Software Engineering
Type of data	Raw Tables (csv)
Data collection	The dataset was curated from Stack Overflow data dump published in October 2025 containing 8 multi-GB XML files namely *Badges, Comments, PostHistory, PostLinks, Posts, Tags, Users* and *Votes*. The large sized XML files were chunked to multiple smaller XML files which were fed to SQL Server database. Once transformed into relational tables, *Questions* and *Answers* were extracted from *Posts* which can originally contain 17 different types of posts including questions and answers. *Questions, Answers* and *Comments* were then applied keywords-based filtering to extract those containing one or more recommendation related terms.
Data source location	The Stack Overflow data dump was downloaded from the Official Stack Overflow site (https://stackoverflow.com/users/data-dump-access/<user-id-of-registered-stackoverflow-user>) as explained on the site’s Help Centre [1].
Data accessibility	Repository name: Stack Overflow Recommendations Dataset (SORD) - Version 1.0 based on Stack Overflow Data Dump - October 2025 Data identification number: 10.6084/m9.figshare.30948506 Direct URL to data: https://doi.org/10.6084/m9.figshare.30948506 Github Repository: https://github.com/arjumandfatima/The-Stack-Overflow-Recommendations-Dataset-SORD-A-Large-Scale-Curated-Recommendations-Dataset-.git
Related research article	Fatima, Arjumand, and Onaiza Maqbool. ``Scalable Conversion of Stack Exchange Network-based Question Answering Sites’ Data Dump to SQL Server Database–A Case of Stack Overflow Data Dump.'' *Array* (2026): 100,793 [2].

## 1 Value of the Data

• **Importance:** To the best of our knowledge, this dataset is one of its kind as the Empirical Software Engineering domain in general and the Recommendation Systems for Software Engineering (RSSE) in particular, lacks relevant data. The dataset is of multidimensional value owing to its scale. The Stack Overflow data dump from which the dataset has been extracted contains historical data from 2008 to 2025 about various technologies containing those that became obsolete, are still in use as legacy, all-time favorite and emerging. While discussing their programming related problems on Stack Overflow, developers not only discuss software or programming languages but several concepts, methodologies, software artifacts, learning resources, technology companies and their products, libraries, frameworks, APIs, tools, platforms and many other entities get discussed. This dataset may contain insightful recommendations related to these entities as well.
  Although, asking for any sort of recommendations on Stack Overflow is considered off topic [3], the extracted dataset can serve as a raw source of crowd wisdom for providing implicit developer recommendations. We found 0.3% of questions asked on the site containing one or more recommendation related keywords that may indicate developers’ quest for some sort of preference or recommendations. Similarly, 6.18% of total answers and 2.08% of total comments contained one or more of these keywords motivating the use of such data for building recommendation systems for software developers.
• **Target Audience**: The dataset is valuable for researchers and practitioners in the field of Natural Language Processing (NLP), Machine Learning (ML), Deep Learning (DL) and Empirical Software Engineering. It is particularly valuable for those interested in RSSE [4] and opinion mining for software engineering. Additionally, researchers interested in analyzing the communication patterns of software developers, being knowledge workers [5], can benefit from this dataset as certain words, terms or expressions may particularly be used in the Software Engineering domain as opposed to common English usage and vice versa.
• **Potential Future Use:** The extracted answers and comments can be mined to extract implicit developer preferences from which ratings can be inferred which can then be utilized to develop multi-criteria software recommendation systems. On the other hand, the extracted recommendation related questions can be transformed into a benchmark for retrieval evaluation of software recommendation systems for developers.
• **Uniqueness of Contribution:** Although extensive literature is available on analyzing Stack Overflow data, studies utilizing a subset of data extracted either from Stack Exchange API or Stack Exchange Data Explorer (SEDE) are more common as compared to longitudinal ones utilizing the complete data dump. Furthermore, owing to the continuously increasing gigantic size of the Stack Overflow data dump, it is becoming increasingly challenging to preprocess the XML based data dump. A handful of longitudinal studies conducted for various kinds of analysis in the past were carried out when the data dump size was relatively smaller and easier to process e.g. for developing an eclipse plugin in 2012 [6] and 2013 [7], for studying the role of emotions expressed in questions [8] and analyzing deleted questions [9] in 2014, for constructing an island grammar in 2015 [10], for studying the evolution of code in 2019 [11] and for summarizing posts at sentence level in 2022 [12]. To the best of our knowledge, no existing large-scale dataset in the software engineering domain, including those derived from Stack Overflow, has been specifically designed and structured for recommendation-oriented tasks. The proposed dataset addresses this gap by providing a comprehensive resource that enables research on recommendation systems and other downstream applications in software engineering. To ensure replicability and transparency, we have not only curated this dataset but also published the procedure utilized for converting the XML based data dump to SQL Server which is a precursor to conducting any such large-scale analysis [2].

## 2 Background

Stack Overflow is a defacto Q&A site for developers to ask programming related questions. However, it considers any sort of recommendation related questions (including books, tutorials, tools, software) as *off topic* as they may attract opinionated, subjective answers and often get closed [3]. However, Stack Exchange Network (hosting Stack Overflow) created a dedicated site for the same purpose namely *Software Recommendations* [13]. Unlike many other popular sites (Stack Overflow [14], Ask Ubuntu [15], Server Fault [16], Super User [17] etc.) it is not widely known and for the last five years it has been the least answered site out of 182 Stack Exchange based Q&A sites with only 57% answered rate. Its presence provides evidence for the need for such software recommendation systems, but its slow answer rate indicates the peculiarity of software engineering domain encompassing its data, information spaces and developers making it more challenging to address recommendation needs of developers [4,18]. With the proliferation of AI tools, the use of chatbots has increased among developers. However, such tools, including the one recently launched by Stack Overflow, namely AI Assist [19], are not well trained to handle such recommendation related queries.

Crowdsourced developer knowledge available on technology related Q&A sites can provide insightful (implicit or explicit) reviews about various aspects of software development that can be utilized to develop different recommenders to facilitate diverse facets of software development lifecycles. Among 71 technology-based Stack Exchange sites (excluding the Russian, Japanese, Spanish and Portuguese variants of Stack Overflow and the Meta Stack Exchange site which deals with meta-discussions about Stack Exchange based Q&A sites), Stack Overflow contained the highest number of tags used by users of Software Recommendations. Hence, it may serve as a source of crowd wisdom for providing implicit or explicit recommendations to developers. The tag overlapping between Software Recommendations and other technology specific sites indicates the potential for utilizing data from such sites to build software recommenders related to various aspects of that technology. The extracted dataset can be helpful for training ML/DL/NLP models in this regard and the systematic approach used for data extraction can be used to extract similar data from other Stack Exchange based technology related Q&A sites.

## 3 Data Description

The data repository comprises of 25 files consisting of 12 csv files containing the curated dataset, 8 csv files containing manually annotated samples used for evaluating false positives, and the remaining 5 are jupyter notebooks for replication of results. The repository is further divided into 6 folders namely questions, answers, comments, additional-metadata, code and evaluation. The questions folder contains 4 csv files; 2 containing resultant records filtered using exact and substring matching applied on question title (i.e. QuestionsTitle_Contain.csv and QuestionsTitle_LikeMinusContain.csv) and the remaining 2 csv files for question body (i.e. QuestionsBody_Contain.csv and QuestionsBody_LikeMinusContain.csv). Similarly, answers folders contain 2 csv files for answers filtered based on exact and substring matching (i.e. Answers_Contain.csv and Answers_LikeMinusContain.csv). Likewise, the comments folder contains 2 csv files namely Comments_Contain.csv and Comments_LikeMinusContain.csv respectively. The code folder contains 3 jupyter notebooks used to (i) convert Stack Overflow data dump to SQL Server database records (StackOverflow_DataDump_Parsers.ipynb), (ii) to curate questions, answers and comments included in SORD from the Stack Overflow data dump (Generating_SORD.ipynb) and (iii) to extract additional metadata associated with SORD (SORD-AdditionalMetadataExtraction.ipynb). Another jupyter notebook (AnalysisofInsightStimulatingExample.ipynb) is included in the package containing the snippets used for analysis done in Section 7. The evaluation folder consists of 8 csv files containing manually annotated sample records extracted using stratified sampling design described in Section 8 along with a jupyter notebook containing the python script used to extract sample data (EstimatingFalsePositivesInSORDUsingStratifiedSamplingStrategy.ipynb).

As answers are associated with questions, they do not have a title of their own. Hence, in case of questions folder there are 2 csv files corresponding to question title which are missing in case of answers which only have body. Similarly, comments are associated with posts (i.e. either question or answer). Hence, they only have a text field but no title. Instead of naming the csv files present in answers as AnswersBody_Contain.csv and AnswersBody_LikeMinusContain.csv, we have simply named them Answers_Contain.csv and Answers_LikeMinusContain.csv. Similarly, in case of comments instead of naming them CommentsText_Contain.csv and CommentsText_LikeMinusContain.csv we have simply named them Comments_Contain.csv and Comments_LikeMinusContain.csv.

While including the filtered records extracted from questions, answers and comments based on keywords, instead of providing all records extracted from substring matching (e.g. instead of producing Comments_Like.csv) we provided the subset of records which were not present in exact matching (i.e. Comments_LikeMinusContain.csv) as substring matching can include exact matching, prefix matching and suffix matching. Providing all records in case of substring matching (including exact matching) would have only added redundancy in the dataset causing greater dataset size.

As SORD is curated from Stack Overflow data dump which follows the Stack Exchange database schema [20], the same is applicable for SORD as well. However, to avoid redundancy, certain columns have been excluded from each of the 12 csv files included in the dataset. We briefly describe the schema followed by Stack Overflow data dump (posts and comments) and compare it with that of SORD followed by the description of data included in SORD.

### 3.1 Schema description

As the dataset contains curated questions, answers and comments extracted from the Stack Overflow data, to understand the structure of the curated dataset, we first need to have a basic understanding of *Comments* and *Posts* (from which questions and answers are extracted). Although Section 4.1 provides a detailed overview of Stack Overflow data dump from which we have extracted SORD, we have provided detailed information about Posts and Comments in Table 1, Table 2, Box 1, Box 2, Box 3, Box 4, Box 5 in this section to make it easy for readers to understand the dataset provided in the repository. Table 1 provides name for each attribute present in the Stack Overflow Posts table along with its data type and description. As posts table may contain different types of posts (including but not limited to questions and answers), different attributes may or may not provide information related to questions and answers. In the next three columns of Table 1 we identify the attributes particularly relevant to questions and answers and mark the presence or absence of each of these attributes in the curated (SORD) dataset’s repository in the relevant columns. Table 2 is simpler as compared to Table 1 which lists each attribute of Stack Overflow columns table along with its datatype and description followed by an indication of whether the attribute is included in relevant csv files of the curated dataset or not.

**Table 1:** Detailed Information of a Stack Overflow Post and Relevant Information Included in SORD.

No	Attribute	Data type	Description	Included in relevant csv files in SORD		
				QuestionsTitle	QuestionsBody	Answers
1	Id	int	ID of the post	✓	✓	✓
2	PostTypeId	tinyint	Type of post (1 represents questions, 2 represents answers and other values for different types of posts up to 17)	**×** (1 for all Qs.^1^)		**×** (2 for all Ans.^2^)
3	AcceptedAnswerId	int	ID of the accepted answer post (optional and value only appears when PostTypeId==1)	✓	✓	**×** (0 for all Ans.)
4	ParentId	int	ID of question post associated with this post (optional and value only appears when PostTypeId==2)	**×** (0 for all Qs.)		✓
5	CreationDate	datetime	Creation date of the post	✓	✓	✓
6	DeletionDate	datetime	Deletion date of the post (date for this column does not exist in data dump)	**×** (Null for all Qs.)		**×** (Null for all Ans.)
7	Score	int	The number of upvotes on a question/answer minus the number of downvotes	✓	✓	✓
8	ViewCount	int	Total number of views for the post (optional and value only appears when PostTypeId==1)	✓	✓	**×** (0 for all Ans.)
9	Body	nvarchar (max)	Body of the post	✓	✓	✓
10	OwnerUserId	int	ID of the post owner (optional and value only appears if the user has not been deleted)	✓	✓	✓
11	OwnerDisplayName	nvarchar (40)	Display name of the post owner (optional and value appears if user has been deleted)	✓	✓	✓
12	LastEditorUserId	int	ID of the user who most recently edited the post (optional)	✓	✓	✓
13	LastEditorDisplayName	nvarchar (40)	Display name of the user who most recently edited the post (optional)	✓	✓	✓
14	LastEditDate	datetime	The date and time of the most recent edit to the post (optional)	✓	✓	✓
15	LastActivityDate	datetime	The date and time of the most recent activity on the post	✓	✓	✓
16	Title	nvarchar (250)	Title of the post in case of question (optional and value appears in cases when PostTypeId is 1,4, or 5)	✓	✓	**×** (Empty for all Ans.)
17	Tags	nvarchar (4000)	Tags associated with question (optional and value only appear in cases when PostTypeId is 1,4, or 5)	✓	✓	**×** (Empty for all Ans.)
18	AnswerCount	int	Number of answers for the post (optional and value appears when PostTypeId=1)	✓	✓	**×** (0 for all Ans.)
19	CommentCount	int	Number of comments on the post	✓	✓	✓
20	FavoriteCount	int	Number of people who like the post (optional and value appears only when PostTypeId==1)	**×** (Null for all Qs.)		**×** (Null for all Ans.)
21	ClosedDate	datetime	The date and time on which the post was closed (optional and appears only the post is closed)	✓	✓	✓
22	CommunityOwnedDate	datetime	Optional and only appears if post is community wiki’d	✓	✓	✓
23	ContentLicense	varchar (30)	The type of license applicable	✓	✓	✓

1 Qs. = Questions.

2 Ans. = Answers.

**Table 2:** Detailed Information of a Stack Overflow Comment and Relevant Information Included in SORD.

No	Attribute	Data type	Description	Included in relevant csv files in SORD (Comments)
1	Id	Int	ID of the comment	✓
2	PostId	Int	ID of the post to which the comment belongs	✓
3	CreationDate	datetime	Creation date of the comment	✓
4	Score	Int	The number of upvotes on a comment minus the number of downvotes	✓
5	Text	nvarchar (600)	Body of the comment	✓
6	UserId	Int	ID of the comment owner	✓
7	UserDisplayName	nvarchar (40)	Display name of the comment owner	✓
8	ContentLicense	varchar (30)	The type of license applicable	✓

### 3.2 Data Description

A brief description of each of the 25 files included in the data repository is given below. For each of the 12-csv files containing the dataset, a sample record is given below its description. Although, the dataset is distributed in standard CSV format, to ensure readability we have presented them here in XML-style format.

**1. \questions\QuestionsTitle_Contain.csv**

It contains all the questions extracted through the full text search applied on the complete set of questions asked on Stack Overflow containing one or more of the keywords in the question title.

**Box 1:** Sample Record from QuestionsTitle_Contain.csv.

<row AutoIdPK="84,413" Id="145,402" AcceptedAnswerId="145,417" CreationDate="2008–09–28 07:09:00.713″ Score="11″ ViewCount="10,551" Body="<p>Can anyone recommend some useful performance analysis tools for PHP scripts? Anything that could help me find problematic or unusually slow blocks of code, details about execution time, etc. would be really helpful. I know there are tools out there, but I'm wondering what people recommend as being the most useful and well-designed.</p>" OwnerUserId="5291″ OwnerDisplayName="Wilco" LastEditorUserId="1033,581" LastEditorDisplayName="Blair Conrad" LastEditDate="2017–04–04 10:23:11.867″ LastActivityDate="2017–04–04 10:23:11.867″ Title="**Can you **recommend** Performance Analysis tools for PHP?**" Tags="<php><performance><testing>" AnswerCount="5″ CommentCount="0″ ClosedDate="2012–06–13 13:29:40.483″ CommunityOwnedDate="1970–01–01 00:00:00.000″ ContentLicense="CC BY-SA 3.0" />

**Box 1:** Sample Record from QuestionsTitle_Contain.csv.

**2. \questions\QuestionsBody_Contain.csv**

It contains all the questions extracted through the full text search applied on the complete set of questions asked on Stack Overflow containing one or more of the keywords in the question body.

**Box 2:** Sample Record from QuestionsBody_Contain.csv.

<row AutoIdPK=``8009” Id=``17,817'' AcceptedAnswerId=``17,822'' CreationDate=``2008–08–20 12:12:13.073” Score=``14” ViewCount=``8858'' Body=``<p>Any **recommendations** for a javascript form validation library. I could try and roll my own (but I'm not very good at javascript). Needs to **support** checking for required fields, and preferably regexp validation of fields.</p>'' OwnerUserId=``1912” OwnerDisplayName=`` robintw'' LastEditorUserId=``362,006'' LastEditorDisplayName="" LastEditDate=``2012–01–02 12:40:24.580” LastActivityDate=``2018–03–22 00:02:56.903” Title=``** Recommendation for javascript form validation library?**'' Tags=``<javascript><forms><validation>'' AnswerCount=``3” CommentCount=``0” ClosedDate=``2013–08–30 08:41:38.207” CommunityOwnedDate=``1970–01–01 00:00:00.000” ContentLicense=`` CC BY-SA 2.5'' />

**Box 2**. Sample Record from QuestionsBody_Contain.csv.

**3. \questions\QuestionsTitle_LikeMinusContain.csv**

It contains all the questions extracted through wildcard substring matching using the SQL LIKE keyword containing one or more of the keywords in the question title but not included in the QuestionsTitle_Contain.csv file. This set may contain false positive or negative results as compared to those included in QuestionsTitle_Contain.csv.

**Box 3:** 

<row AutoIdPK="36,341" Id="67,200" AcceptedAnswerId="0" CreationDate="2008–09–15 21:31:13.297″ Score="3″ ViewCount="3525" Body="<p>We have a project with over 500,000 lines of VB.NET that we need to convert to C#. Any recommendations, based on experience, for tools to use? We are using Visual Studio 2008 and we're targeting 3.5 .</p>" OwnerUserId="10,104" OwnerDisplayName=" Mike" LastEditorUserId="0″ LastEditorDisplayName="" LastEditDate="1970–01–01 00:00:00.000″ LastActivityDate="2018–01–24 22:34:42.563″ Title=" Tool **recommendation** for converting VB to C#" Tags="<c#><vb.net><visual-studio-2008>" AnswerCount="7″ CommentCount="2″ ClosedDate="2013–07–23 21:13:36.993″ CommunityOwnedDate="1970–01–01 00:00:00.000″ ContentLicense="CC BY-SA 2.5" />

**Box 3** Sample Record from QuestionsTitle_LikeMinusContain.csv.

**4 \questions\QuestionsBody_LikeMinusContain.csv**

It contains all the questions extracted through wildcard substring matching using the SQL LIKE keyword containing one or more of the keywords in the question body but not included in the QuestionsBody_Contain.csv file. This set may contain false positive or negative results as compared to those included in QuestionsBody_Contain.csv.

**Box 4:** 

<row AutoIdPK="33,031" Id="61,972" AcceptedAnswerId="61,987" CreationDate="2008–09–15 07:28:56.910″ Score="46″ ViewCount="84,948" Body="<p>Best **recommendations** for accessing and manipulation of sqlite databases from JavaScript.</p>" OwnerUserId="6390″ OwnerDisplayName="" LastEditorUserId="" LastEditorDisplayName="" LastEditDate="1970–01–01 00:00:00.000″ LastActivityDate="2017–07–19 11:47:12.830″ Title="JavaScript sqlite" Tags="<javascript><sqlite>" AnswerCount="10″ CommentCount="4″ ClosedDate="2017–07–19 13:45:34.433″ CommunityOwnedDate="1970–01–01 00:00:00.000″ ContentLicense=" CC BY-SA 2.5" />

**Box 4:** Sample Record from QuestionsBody_LikeMinusContain.csv.

**5. \answers\Answers_Contain.csv**

It contains all the answers extracted through the full text search applied on the complete set of answers posted on Stack Overflow containing one or more of the keywords in the answer body.

**Box 5:** 

<row AutoIdPK="28,424,230" Id="35,675,250" ParentId="35,674,143" CreationDate="2016–02–27 20:47:07.450″ Score="0" Body="<p>There is a free library <strong>ImgSource</strong> that you can use which has a huge amount of functionality for images.</p><p><a href="http://www.smalleranimals.com/isource.htm" rel="nofollow">http://www.smalleranimals.com/isource.htm</a></p><p>It is available in MSDN, but using a library like <strong>ImgSource</strong> may be to your advantage.</p><p>It provides simple methods for rendering your image onto a device context. And there is a good **support** forum.</p>" OwnerUserId="2287,576" OwnerDisplayName="" LastEditorUserId="0″ LastEditorDisplayName="" LastEditDate="1970–01–01 00:00:00.000″ LastActivityDate="2016–02–27 20:47:07.450″ CommentCount="0″ ClosedDate="1970–01–01 00:00:00.000″ CommunityOwnedDate="1970–01–01 00:00:00.000″ ContentLicense="CC BY-SA 3.0" />

**Box 5:** Sample Record from Answers_Contain.csv.

**6. \answers\Answers_LikeMinusContain.csv**

It contains all the answers extracted through wildcard substring matching using the SQL LIKE keyword containing one or more of the keywords in the answer body but not included in the Answers_Contain.csv file. This set may contain false positive or negative results as compared to those included in Answers_Contain.csv.

**7. \comments\Comments_Contain.csv**

**Table:** 

<row AutoIdPK="14,753" Id="31,239" ParentId="31,215" CreationDate="2008–08–27 20:46:07.167″ Score="1" Body="<p>As has been **suggested**, custom types is the way to go in this case.</p>" OwnerUserId="2284″ OwnerDisplayName="17 of 26″ LastEditorUserId="0″ LastEditorDisplayName="" LastEditDate="1970–01–01 00:00:00.000″ LastActivityDate="2008–08–27 20:46:07.167″ CommentCount="0″ ClosedDate="1970–01–01 00:00:00.000″ CommunityOwnedDate="1970–01–01 00:00:00.000″ ContentLicense="CC BY-SA 2.5" />

**Box 6:** Sample Record from Answers_LikeMinusContain.

It contains all the comments extracted through the full text search applied on the complete set of comments posted on Stack Overflow containing one or more of the keywords in the comments text.

**Table:** 

<row AutoIdPK=``21,229'' Id=``8406,136'' PostId=``97,922'' Score=``1” Text=``On the download page there is the portable version: http://code.google.com/p/msysgit/downloads/list Currently the latest version says ``beta'' but it is very stable. I am using it a lot with no issues. Can absolutely **recommend** it.'' CreationDate=``2011–08–12 02:35:07.837” UserDisplayName =``0” UserId =``362,951'' ContentLicense=`` CC BY-SA 3.0'' />

**Box 7:** Sample Record from Comments_Contain.csv.

**8. \comments\Comments_LikeMinusContain.csv**

It contains all the comments extracted through wildcard substring matching using the SQL LIKE keyword containing one or more of the keywords in the comment text but not included in the Comments_Contain.csv file. This set may contain false positive or negative results as compared to those included in Comments_Contain.csv.

**Table:** 

<row AutoIdPK="1718,792" Id="2067,847" PostId="2129,051" Score="1″ Text=" My **recommendation**: *don't*. At least, not at first. Learn MIPS or SPARC first to get your head around assembly, and then go for the more complex architecture." CreationDate="2010–01–24 22:06:27.420″ UserDisplayName ="0″ UserId ="224,346" ContentLicense="CC BY-SA 2.5" />

**Box 8:** Sample Record from Comments_LikeMinusContain.csv.

**9. \additional-metadata\FilteredBadges.csv**

It contains all the badges associated with Users (through UserId) who posted the questions, answers or comments present in the above-mentioned files.

**Table:** 

<row AutoIdPK=``177,546'' Id=``20,549,833'' UserId =``1369,547'' Name=`` Teacher '' Date=``2016–10–10 13:19:54.793” Class=``3” TagBased=``0'' />

**Box 9:** Sample Record from FilteredBadges.csv.

**10 \additional-metadata\FilteredTags.csv**

It contains all the tags associated with Posts (questions, answers or parent post of comments) that are included in the above-mentioned files.

**Table:** 

<row Tag=".net" Count="111,222" />

**Box 10:** Sample Record from FilteredTags.csv.

**11 \additional-metadata\FilteredVotes.csv**

It contains all the votes associated with Posts (questions, answers or parent post of comments) that are included in the above-mentioned files.

**Table:** 

<row AutoIdPK="629,232" Id="306,389" PostId ="2742″ VoteTypeId="2″ UserId="NULL" CreationDate="2008–09–22 00:00:00.000″ BountyAmount="NULL" />

**Box 11:** Sample Record from FilteredVotes.csv.

**12 \additional-metadata\FilteredUsers.csv**

It contains all the users who have posted the questions, answers or comments that are included in the above-mentioned files.

**Table:** 

<row AutoIdPK="53″ Id="46″ Reputation ="18,398" CreationDate="2008–08–01 13:13:21.413″ DisplayName ="sven" LastAccessDate="2017–05–29 11:08:05.950″ WebsiteUrl ="http://-" Location ="Belgium" AboutMe="0″ Views="1560″ Upvotes="490″ DownVotes="17″ ProfileImageUrl="NULL" AccountId="37"/>

**Box 12:** Sample Record from FilteredUsers.csv.

**1. \code\StackOverflow_DataDump_Parsers.ipynb**

It contains detailed instructions on converting the data dump (8 XML files) to corresponding database tables in SQL Server. This responds to the data preprocessing step explained in Section 4.1. A more detailed explanation of this preprocessing step is also available for those interested in replicating this study or preprocessing the data dump of any Stack Exchange site (including Stack Overflow) for any kind of longitudinal analysis [2].

**2. \code\Generating_SORD.ipynb**

It contains information and instructions about the keyword-based extraction process explained in Section 4.3.

**3. \code\SORD-AdditionalMetadataExtraction.ipynb**

It contains information regarding the extraction of additional metadata including Badges, Tags, Users, and Votes respectively as explained in Section 4.4.

**4. \AnalysisofInsightStimulatingExample.ipynb**

It contains information regarding the analysis of a sample usecase done in Section 7.

**5. \evaluation\**

This folder contains 8 csv files containing stratified samples that were manually annotated either as true positive or false positive for estimating the false positive rate and precision as described in Section 8. It also contains EstimatingFalsePositivesInSORDUsingStratifiedSamplingStrategy.ipynb which has the python script used to extract the stratified samples from SORD.

## 4 Experimental Design, Materials and Methods

**Fig. 1:**
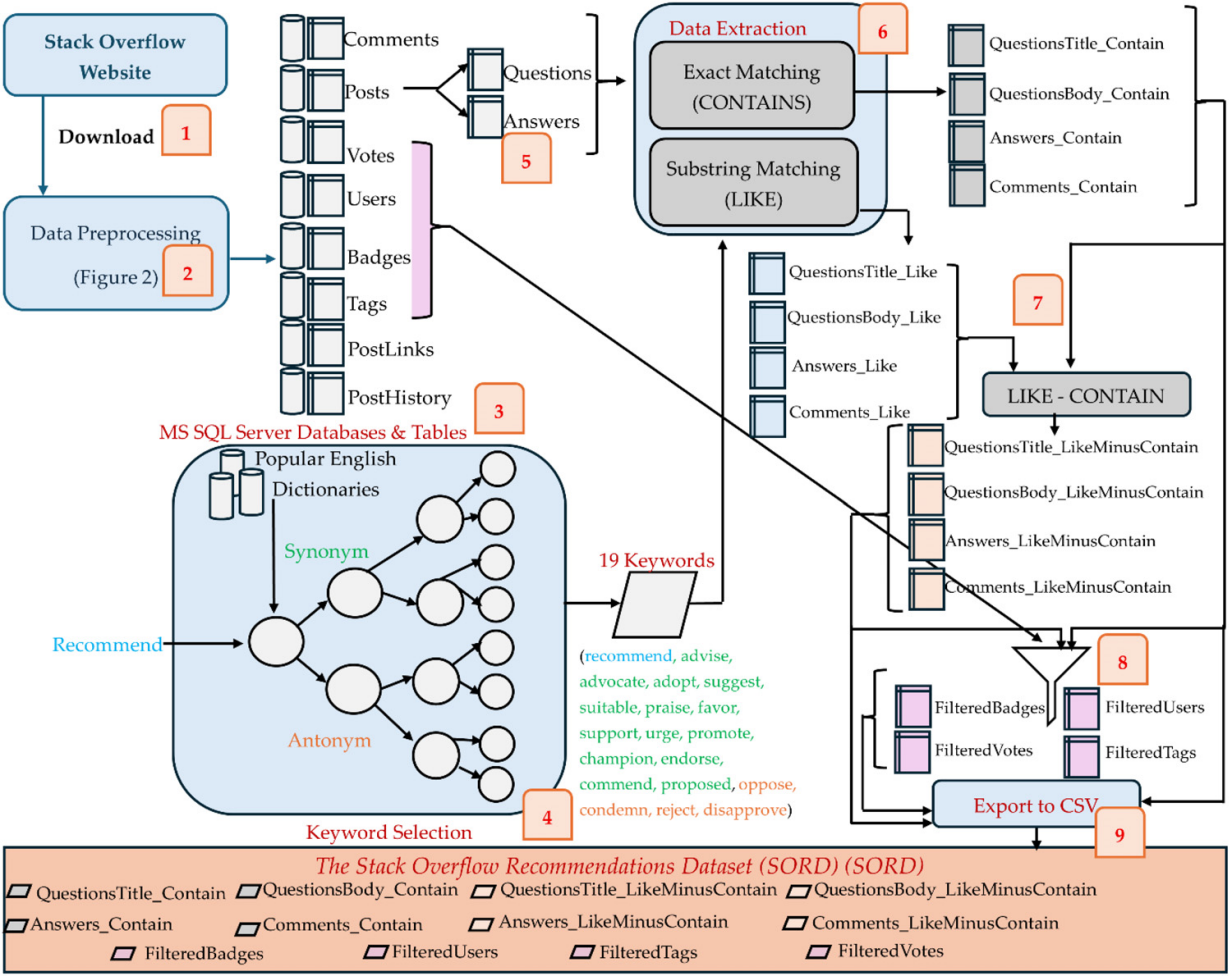
Complete Workflow for Curating SORD.

**Fig. 2:**
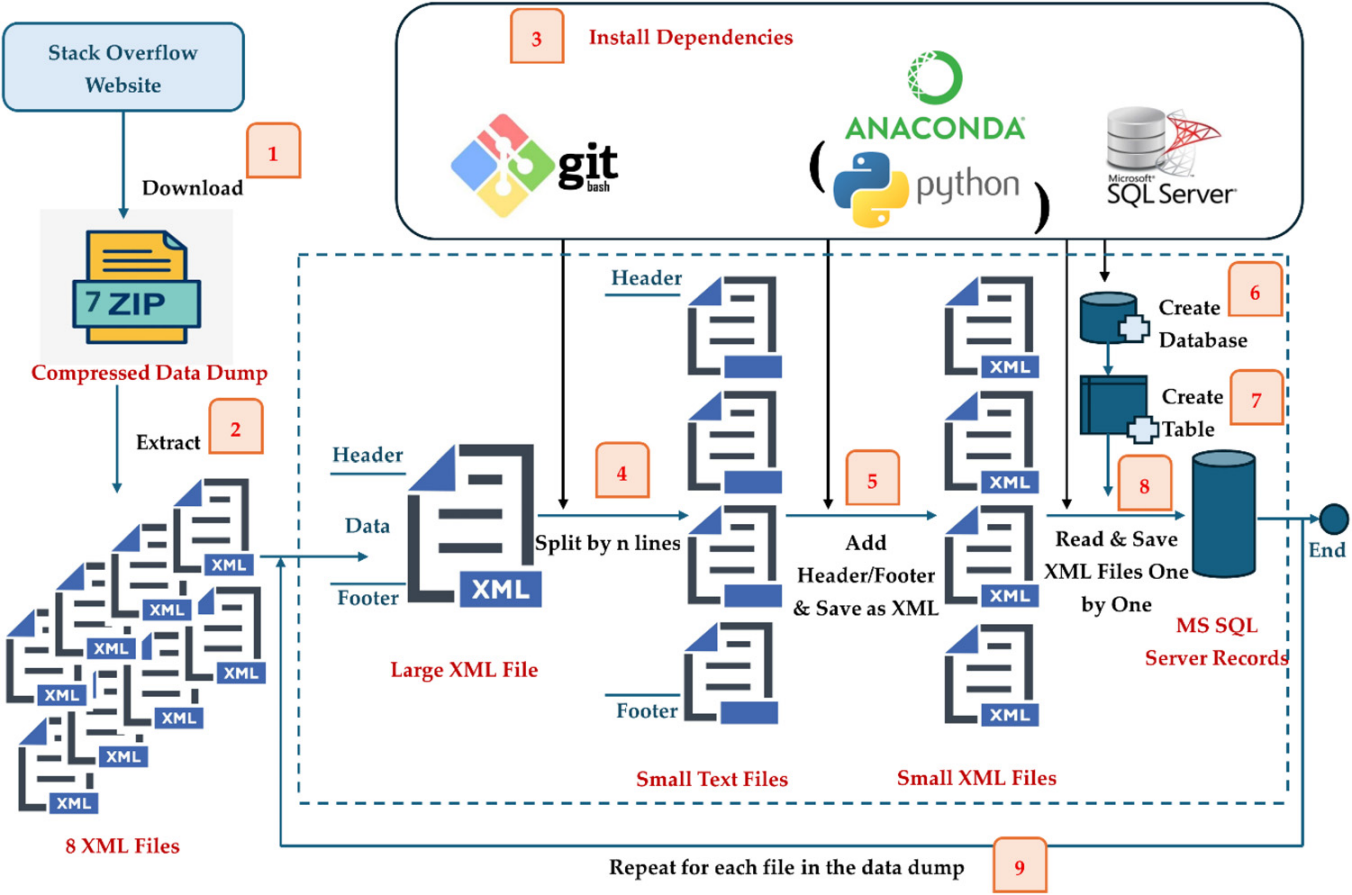
Preprocessing of XML Based Stack Overflow Data Dump.

This section describes the *S*tack *O*verflow *R*ecommendations *D*ataset (*SORD*) and provides an overview of the approach used to curate it. Fig. 1 provides a holistic overview of steps followed to curate SORD from Stack Overflow data dump. The curation of SORD stated from downloading of XML based Stack Overflow data dump from its official website [1] (step 1) which was converted to MS SQL Server database records (step 2 and 3) which is explained in detail in Fig. 2. A set of keywords were selected using seeded snowball sampling method based on their dictionary meaning (step 4). Questions and answers were extracted from Posts (step 5) and then keywords-based data extraction was performed on questions, answers and comments using exact matching and substring matching (step 6). As substring matching can contain the results of exact matching as subset, we excluded the redundant records from substring matching result set (step 7). After extracting the core part of SORD, we extracted additional metadata about badges, tags, users and votes (step 8). Each of these steps demonstrated in Fig. 1 are explained in more detail in the subsequent subsections.

### 4.1 Data preprocessing

We downloaded the Stack Overflow data dump published in October 2025 from the official Stack Overflow website [1]. The data dump is provided as 7zip compressed folder containing 8 XML files. The size of each XML file (after extraction of the compressed folder) is given in Table 3 along with the total number of records saved in the corresponding MS SQL Server tables because of data preprocessing step. Fig. 2 demonstrates the steps involved in converting the XML based Stack Overflow data dump to MS SQL Server database records. A detailed description of the dataset generation workflow is provided in a separate methodology manuscript [2] as the same can be used for preprocessing the data dump of any of the existing 182 Stack Exchange Network based Q&A sites. This can itself be useful for the researchers and practitioners interested in utilizing data from any of these sites particularly at a large scale which cannot be done otherwise through Stack Exchange Data Explorer or Stack Exchange API due to their usage restrictions. The same is covered in \code\StackOverflow_DataDump_Parsers.ipynb for the sake of completeness for the readers here.

Owing to the gigantic size of the XML files except *Tags* and *PostLinks*, it was difficult to process them in main memory directly so we divided each of the large sized XML files into smaller chunks using Git Bash’s split command (split by lines) [2,21] (step 4 in Fig. 2). Proper XML header/footer was appended to each chunk to make it a valid XML file which can be easily processed (step 5 in Fig. 2). The XML records were then stored in corresponding SQL Server database tables for further querying (step 8 in Fig. 2). Sample XML records corresponding to each of the 8 files described in Table 3 are given in Table 4. The extracted text fields were not subjected to any additional normalization or cleaning steps. HTML entities, code blocks, and escape characters were preserved as-is from the original Stack Overflow data dump to ensure fidelity and reproducibility of the dataset.

**Table 3:** Overview of Stack Overflow Data Dump (October 2025).

File Name	File Size	No. of Records After Parsing
Badges	5.99 GB	54,312,436 (54 M)
Comments	26.2 GB	91,315,031 (91 M)
PostHistory	172 GB	162,338,606 (162 M)
PostLinks	767 MB	6535,632 (6.5 M)
Posts	98.4 GB	60,376,057 (60 M)
Tags	5.60 MB	66,046 (66k)
Users	7.25 GB	30,088,388 (30 M)
Votes	22.3 GB	246,514,372 (247 M)

**Table 4:** Sample XML Records from Stack Overflow Data Dump.

File Name	Sample XML Record
Badges	<row Id="82,957" UserId="5246″ Name="Teacher" Date="2008–09–15T08:55:03.957″ Class="3″ TagBased="False" />
Comments	<row Id="18,574,249" PostId="13,558,297" Score="0″ Text="hey bro, i tried this code, and unfortunately its doing the same thing, on the second ‘NSLog‘, its returning ‘NULL‘.:(ive done this a million times before. But this time is giving me a hard time! Thank you for the help!" CreationDate="2012–11–26T03:55:06.310″ UserId="1784,502" ContentLicense="CC BY-SA 3.0" />
PostHistory	<row Id="932,798" PostHistoryTypeId="5″ PostId="556,898" RevisionGUID="bc5d6b08–94b6–43a6-bc06-b5e450f8c0df" UserId="20,860" CreationDate="2009–02–17T14:18:00.017″ ContentLicense="CC BY- SA 2.5″ Comment="deleted 5 characters in body" Text="Yes, a primary key is not only good practice – it’s crucial. A table that lacks a unique key fails to be in [First Normal Form] [2]. You must declare a PRIMARY KEY or UNIQUE constraint if you want other tables to reference this one with a foreign key.In most RDBMS brands, both PRIMARY KEY and UNIQUE constraints implicitly create an index on the column(s). If it doesn’t do this implicitly, you may be required to define the index yourself before you can declare the constraint. [2]: http://en.wikipedia.org/wiki/First_normal_form" />
PostLinks	<row Id="244″ CreationDate="2010–04–26T03:00:03.780″ PostId="24,319" RelatedPostId="1711″ LinkTypeId="1" />
Posts	<row Id="3848,243" PostTypeId="2″ ParentId="3848,112" CreationDate="2010–10–03 T01:59:38.713″ Score="0″ Body="&lt; p&gt; You should not be using the shell with &lt;code&gt;&&lt;/code&gt; to run background processes. If you do that, they come out as grandchildren which you cannot track and wait on. Instead you need to either mimic what the shell does to run background processes in your own code, or it would probably work just as well to close the terminal (or rather stdin/out/err) and open &lt;code&gt;/dev/null&lt;/code&gt; in its place in the child processes so they don’t try to write to the terminal or take control of it.&lt;/p&gt;" OwnerUserId="379,897" LastActivityDate=" 2010–10–03T01:59:38.713″ CommentCount="3″ ContentLicense="CC BY-SA 2.5"/>
Tags	<row Id="155,127" TagName="quickchart" Count="1″ ExcerptPostId="71,287,072" WikiPostId="71,287,071" />
Users	<row Id="22,668" Reputation="3425″ CreationDate="2008–09–26T12:41:24.470″ DisplayName="Dickon Reed" LastAccessDate="2018–01–20T13:33:15.500″ WebsiteUrl="" Location="Cambridge, United Kingdom" AboutMe="" Views="663″ UpVotes="89″ DownVotes="3″ AccountId="11,685" />
Votes	<row Id="42,569,075" PostId="2400″ VoteTypeId="2″ CreationDate="2013–03–05T00:00:00.000" />

### 4.2 Keywords selection

We started with the term ‘recommend’ and tried to analyze its meaning mentioned in different dictionaries online [[22], [23], [24], [25]]. Based on its meaning we curated a list of synonyms and antonyms mentioned in Table 5. The keyword selection approach can be considered analogous to the seeded snowball sampling technique whereby the term ‘recommend’ was used as a seed.

**Table 5:** Keywords and their meanings.

Keyword	Meaning
Recommend	− to suggest that someone or something would be good or suitable for a particular job or purpose, or to suggest that a particular action should be done − to advise someone to do something − to suggest that something is the best thing to choose − to present (something) as worthy of acceptance or trial − to endorse (someone) as fit, worthy, or competent − to suggest (an act or course of action) as advisable − to advise a particular course of action; to advise somebody to do something − to praise or commend to another as being worthy or desirable; endorse − to make attractive or acceptable − to represent or urge as advisable or expedient − to present as worthy of confidence, acceptance, use etc.; commend; mention favorably
Advise	− to give a recommendation about what should be done
Advocate	− to support or argue for (a cause, policy etc.) − to plead in favor of − to act as advocate for someone or something
Adopt	− to begin to practice or use (something, such as an approach or manner) − to accept and establish (something, such as a law or policy) in a formal or official way
Suggest	− to mention or imply as a possibility − to propose as desirable or fitting − to offer for consideration or as a hypothesis
Suitable	− adapted to a use or purpose − able, qualified
Praise	− an expression of approval − to express a favorable judgement of − to express praise
Favor	− approving consideration or attention − to show partiality towards − to regard or treat with favor
Support	− to argue or vote for − the act or process of supporting − assistance provided by a company to users of its products − to keep (something) going − to uphold or defend as valid or right
Urge	− to force or impel in an indicated direction or into motion or greater speed − to declare, advance, or press earnestly a statement, argument, charge, or claim
Promote	− to contribute to the growth or prosperity of
Champion	− a person who fights for, or speaks in support of, a group of people or a belief − a person, team etc. that has won a competition, especially in a sport
Endorse	− to approve openly − to recommend (something, such as a product or service) usually for financial compensation
Commend	− to recommend as worthy of confidence or notice
Propose	− to set forth for acceptance or rejection − to recommend filling a place or vacancy − to form or put forward a plan or intention
Oppose	− to resist − to place opposite or against something − to place over against something to provide resistance, counterbalance, or contrast
Condemn	− to adjudge unfit for use or consumption − to declare to be reprehensible, wrong or evil usually after weighing evidence and without reservation
Reject	− to refuse to accept, consider, submit to, take for some purpose, or use
Disapprove	− to pass unfavorable judgment on − to refuse approval to − to feel or express disapproval

### 4.3 Data extraction

Before proceeding with this step, we assume that the readers have successfully converted the XML based Stack Overflow data dump to MS SQL Server database records by following the steps mentioned in \code\StackOverflow_DataDump_Parsers.ipynb [2]. From this point onwards, we will be referring to the following SQL Server database tables corresponding to the 8 XML files present in the Stack Overflow data dump (as presented in Table 6).

**Table 6:** SQL Server Tables Corresponding to 8 XML Files Present in Stack Overflow Data Dump.

XML File in Data Dump	SQL Server Database and Table Name
Badges.xml	[StackOverflowBadgesDb_Oct2025].[dbo].[StackOverflowBadges]
Comments.xml	[StackOverflowCommentsDb_Oct2025].[dbo].[StackOverflowComments]
PostHistory.xml	[StackOverflowPostHistoryDb_Oct2025].[dbo].[StackOverflowPostHistory]
PostLinks.xml	[StackOverflowPostLinksDb_Oct2025].[dbo].[StackOverflowPostLinks]
Posts.xml	[StackOverflowPostsDb_Oct2025].[dbo].[StackOverflowPosts]
Tags.xml	[StackOverflowTagsDb_Oct2025].[dbo].[StackOverflowTags]
Users.xml	[StackOverflowUsersDb_Oct2025].[dbo].[StackOverflowUsers]
Votes.xml	[StackOverflowVotesDb_Oct2025].[dbo].[StackOverflowVotes]

It is pertinent to mention here that our methodology for data preprocessing and data extraction/curation is particularly based on MS SQL Server as we have utilized its full text search feature in case of exact matching. We have not evaluated it for other popular database management systems such as MySQL and PostgreSQL. This design choice was particularly made because Stack Exchange itself uses SQL Server for storing the data of all the sites.

The Stack Overflow data dump, provided in XML format, was parsed based on its standard structure without requiring any custom or platform-specific modifications. All filtering and extraction operations were performed using SQL Server queries, and no cross-database compatibility layer (e.g., PostgreSQL or MySQL specific adaptations) was considered in this work. These platform choices ensure a reproducible and straightforward implementation within a single database environment.

The data extraction process revolved around the following two research questions (RQs):

• RQ1: Do developers *look for* any sort of recommendations on Stack Overflow?
• RQ2: Do developers *provide* any sort of recommendations on Stack Overflow?

To answer the first question, we extracted all questions asked on Stack Overflow available in the data dump inside Posts.xml (by considering all the Posts with PostTypeId=1). On the other hand, to answer the second question we considered Stack Overflow answers (extracted from Posts.xml by considering all Posts with PostTypeId=2) and Comments (all records from Comments.xml).

#### 4.3.1 Extracting stack overflow questions and answers from posts

We used the SQL queries given in Box 13 to extract questions and answers from Posts. The comments table mentioned in Table 6 was directly used to extract recommendations related comments.

**Table:** 

**SELECT** * into [StackOverflowPostsDb_Oct2025].[dbo].[StackOverflowQuestions] **FROM** [StackOverflowPostsDb_Oct2025].[dbo].[StackOverflowPosts] **where** PostTypeId**=**1 **SELECT** * into [StackOverflowPostsDb_Oct2025].[dbo].[StackOverflowAnswers] **FROM** [StackOverflowPostsDb_Oct2025].[dbo].[StackOverflowPosts] **where** PostTypeId**=**2

**Box 13:** SQL Queries for Extracting Questions and Answers from Stack Overflow Posts.

#### 4.3.2 Keywords-based filtering of stack overflow questions, answers and comments

After extracting all questions, we applied keywords-based filtering (using our set of keywords mentioned in Table 5)in two ways i.e. exact matching and substring matching. For exact matching, we utilized the SQL Server’s full text search feature which uses the CONTAINS keyword. In case of substring matching, we utilized the SQL LIKE keyword [26,27]. We applied both the techniques to Question Title and Question Body for answering RQ1.

To answer the RQ2, we considered Stack Overflow Answers (extracted from Posts.xml by considering all Posts with PostTypeId=2) and Comments (all records from Comments.xml). We again applied both exact and substring matching as described above using the same set of keywords mentioned in Table 5 and compared the results obtained from both methods. The SQL queries used for exact matching using SQL Server’s full text search feature are given in Box 14 while the queries for substring matching using wild-card searching are given in Box 15. The replication package contains instructions on creating the corresponding databases and tables in SQL Server and indexes for each table required for using the full text feature. The readers are advised to see the replication package for smooth replication of the stated procedure. As substring matching can contain the results obtained from exact matching, we eliminated the overlapping records by using the SQL queries given in Box 16. The results obtained for the above-mentioned research questions for both the methods are presented in Table 7.

**Box 14:** Exact Matching Using Full Text Search feature and CONTAIN keyword to Extract Recommendations Related Questions, Answers and Comments.

**Filter *Questions* that contain the (exact) keywords in *Title***
DECLARE @keywords NVARCHAR(MAX) = '``recommend'' OR ``advise'' OR ``advocate'' OR ``adopt'' OR ``suggest'' OR ``suitable'' OR ``praise'' OR ``favor'' OR ``support'' OR ``urge'' OR ``promote'' OR ``champion'' OR ``endorse'' OR ``commend'' OR ``propose'' OR ``oppose'' OR ``condemn'' OR ``reject'' OR ``disapprove'''; DECLARE @sql NVARCHAR(MAX); SET @sql = ' SELECT * INTO [SORecommendationsDb].[dbo].[QuestionsTitle_Contain] FROM [StackOverflowPostsDb_Oct2025].[dbo].[StackOverflowQuestions] WHERE CONTAINS(Title, ''' + @keywords + '''); '; EXEC sp_executesql @sql;
**Filter *Questions* that contain the (exact) keywords in *Body***
DECLARE @keywords NVARCHAR(MAX) = '``recommend'' OR ``advise'' OR ``advocate'' OR ``adopt'' OR ``suggest'' OR ``suitable'' OR ``praise'' OR ``favor'' OR ``support'' OR ``urge'' OR ``promote'' OR ``champion'' OR ``endorse'' OR ``commend'' OR ``propose'' OR ``oppose'' OR ``condemn'' OR ``reject'' OR ``disapprove'''; DECLARE @sql NVARCHAR(MAX); SET @sql = ' SELECT * INTO [SORecommendationsDb].[dbo].[QuestionsBody_Contain] FROM [StackOverflowPostsDb_Oct2025].[dbo].[StackOverflowQuestions] WHERE CONTAINS(Body, ''' + @keywords + '''); '; EXEC sp_executesql @sql;
**Filter *Answers* that contain the (exact) keywords**
DECLARE @keywords NVARCHAR(MAX) = '``recommend'' OR ``advise'' OR ``advocate'' OR ``adopt'' OR ``suggest'' OR ``suitable'' OR ``praise'' OR ``favor'' OR ``support'' OR ``urge'' OR ``promote'' OR ``champion'' OR ``endorse'' OR ``commend'' OR ``propose'' OR ``oppose'' OR ``condemn'' OR ``reject'' OR ``disapprove'''; DECLARE @sql NVARCHAR(MAX); SET @sql = ' SELECT * INTO [SORecommendationsDb].[dbo].[Answers_Contain] FROM [StackOverflowPostsDb_Oct2025].[dbo].[StackOverflowAnswers] WHERE CONTAINS(Body, ''' + @keywords + '''); '; EXEC sp_executesql @sql;
**Filter *Comments* that contain the (exact) keywords**
DECLARE @keywords NVARCHAR(MAX) = '``recommend'' OR ``advise'' OR ``advocate'' OR ``adopt'' OR ``suggest'' OR ``suitable'' OR ``praise'' OR ``favor'' OR ``support'' OR ``urge'' OR ``promote'' OR ``champion'' OR ``endorse'' OR ``commend'' OR ``propose'' OR ``oppose'' OR ``condemn'' OR ``reject'' OR ``disapprove'''; DECLARE @sql NVARCHAR(MAX); SET @sql = ' SELECT * INTO [SORecommendationsDb].[dbo].[Comments_Contain] FROM [StackOverflowCommentsDb_Oct2025].[dbo].[StackOverflowComments] WHERE CONTAINS(Text, ''' + @keywords + '''); '; EXEC sp_executesql @sql;

**Box 15:** Substring Matching Using Wildcard Search with LIKE keyword to Extract Recommendations Related Questions, Answers and Comments.

**Filter *Questions* that contain the keywords (as substrings) in *Title***
**DECLARE @sql** NVARCHAR(**MAX**); **DECLARE @**columnName sysname **=** 'Title'; **DECLARE @**likeKeywords NVARCHAR(**MAX**) **=** **@**columnName **+** ' LIKE ''%recommend%'' OR ' **+** **@**columnName **+** ' LIKE ''%advise%'' OR ' **+** **@**columnName **+** ' LIKE ''%advocate%'' OR ' **+** **@**columnName **+** ' LIKE ''%adopt%'' OR ' **+** **@**columnName **+** ' LIKE ''%suggest%'' OR ' **+** **@**columnName **+** ' LIKE ''%suitable%'' OR ' **+** **@**columnName **+** ' LIKE ''%praise%'' OR ' **+** **@**columnName **+** ' LIKE ''%favor%'' OR ' **+** **@**columnName **+** ' LIKE ''%support%'' OR ' **+** **@**columnName **+** ' LIKE ''%urge%'' OR ' **+** **@**columnName **+** ' LIKE ''%promote%'' OR ' **+** **@**columnName **+** ' LIKE ''%champion%'' OR ' **+** **@**columnName **+** ' LIKE ''%endorse%'' OR ' **+** **@**columnName **+** ' LIKE ''%commend%'' OR ' **+** **@**columnName **+** ' LIKE ''%propose%'' OR ' **+** **@**columnName **+** ' LIKE ''%oppose%'' OR ' **+** **@**columnName **+** ' LIKE ''%condemn%'' OR ' **+** **@**columnName **+** ' LIKE ''%reject%'' OR ' **+** **@**columnName **+** ' LIKE ''%disapprove%'''; **SET @sql =** ' SELECT * INTO [SORecommendationsDb].[dbo].[QuestionsTitle_Like] FROM [StackOverflowPostsDb_Oct2025].[dbo].[StackOverflowQuestions] WHERE ' **+ @**likeKeywords **+** '; '; **EXEC** sp_executesql **@sql**;
**Filter *Questions* that contain the keywords (as substrings) in *Body***
**DECLARE @sql** NVARCHAR(**MAX**); **DECLARE @**columnName sysname **=** 'Body'; **DECLARE @**likeKeywords NVARCHAR(**MAX**) **=** **@**columnName **+** ' LIKE ''%recommend%'' OR ' **+** **@**columnName **+** ' LIKE ''%advise%'' OR ' **+** **@**columnName **+** ' LIKE ''%advocate%'' OR ' **+** **@**columnName **+** ' LIKE ''%adopt%'' OR ' **+** **@**columnName **+** ' LIKE ''%suggest%'' OR ' **+** **@**columnName **+** ' LIKE ''%suitable%'' OR ' **+** **@**columnName **+** ' LIKE ''%praise%'' OR ' **+** **@**columnName **+** ' LIKE ''%favor%'' OR ' **+** **@**columnName **+** ' LIKE ''%support%'' OR ' **+** **@**columnName **+** ' LIKE ''%urge%'' OR ' **+** **@**columnName **+** ' LIKE ''%promote%'' OR ' **+** **@**columnName **+** ' LIKE ''%champion%'' OR ' **+** **@**columnName **+** ' LIKE ''%endorse%'' OR ' **+** **@**columnName **+** ' LIKE ''%commend%'' OR ' **+** **@**columnName **+** ' LIKE ''%propose%'' OR ' **+** **@**columnName **+** ' LIKE ''%oppose%'' OR ' **+** **@**columnName **+** ' LIKE ''%condemn%'' OR ' **+** **@**columnName **+** ' LIKE ''%reject%'' OR ' **+** **@**columnName **+** ' LIKE ''%disapprove%'''; **SET @sql =** ' SELECT * INTO [SORecommendationsDb].[dbo].[QuestionsBody_Like] FROM [StackOverflowPostsDb_Oct2025].[dbo].[StackOverflowQuestions] WHERE ' **+ @**likeKeywords **+** '; '; **EXEC** sp_executesql **@sql**;
**Filter *Answers* that contain the keywords (as substrings)**
**DECLARE @sql** NVARCHAR(**MAX**); **DECLARE @**columnName sysname **=** 'Body'; **DECLARE @**likeKeywords NVARCHAR(**MAX**) **=** **@**columnName **+** ' LIKE ''%recommend%'' OR ' **+** **@**columnName **+** ' LIKE ''%advise%'' OR ' **+** **@**columnName **+** ' LIKE ''%advocate%'' OR ' **+** **@**columnName **+** ' LIKE ''%adopt%'' OR ' **+** **@**columnName **+** ' LIKE ''%suggest%'' OR ' **+** **@**columnName **+** ' LIKE ''%suitable%'' OR ' **+** **@**columnName **+** ' LIKE ''%praise%'' OR ' **+** **@**columnName **+** ' LIKE ''%favor%'' OR ' **+** **@**columnName **+** ' LIKE ''%support%'' OR ' **+** **@**columnName **+** ' LIKE ''%urge%'' OR ' **+** **@**columnName **+** ' LIKE ''%promote%'' OR ' **+** **@**columnName **+** ' LIKE ''%champion%'' OR ' **+** **@**columnName **+** ' LIKE ''%endorse%'' OR ' **+** **@**columnName **+** ' LIKE ''%commend%'' OR ' **+** **@**columnName **+** ' LIKE ''%propose%'' OR ' **+** **@**columnName **+** ' LIKE ''%oppose%'' OR ' **+** **@**columnName **+** ' LIKE ''%condemn%'' OR ' **+** **@**columnName **+** ' LIKE ''%reject%'' OR ' **+** **@**columnName **+** ' LIKE ''%disapprove%'''; **SET @sql =** ' SELECT * INTO [SORecommendationsDb].[dbo].[Answers_Like] FROM [StackOverflowPostsDb_Oct2025].[dbo].[StackOverflowAnswers] WHERE ' **+ @**likeKeywords **+** '; '; **EXEC** sp_executesql **@sql**;
**Filter *Comments* that contain the keywords (as substrings)**
**DECLARE @sql** NVARCHAR(**MAX**); **DECLARE @**columnName sysname **=** 'Text'; **DECLARE @**likeKeywords NVARCHAR(**MAX**) **=** **@**columnName **+** ' LIKE ''%recommend%'' OR ' **+** **@**columnName **+** ' LIKE ''%advise%'' OR ' **+** **@**columnName **+** ' LIKE ''%advocate%'' OR ' **+** **@**columnName **+** ' LIKE ''%adopt%'' OR ' **+** **@**columnName **+** ' LIKE ''%suggest%'' OR ' **+** **@**columnName **+** ' LIKE ''%suitable%'' OR ' **+** **@**columnName **+** ' LIKE ''%praise%'' OR ' **+** **@**columnName **+** ' LIKE ''%favor%'' OR ' **+** **@**columnName **+** ' LIKE ''%support%'' OR ' **+** **@**columnName **+** ' LIKE ''%urge%'' OR ' **+** **@**columnName **+** ' LIKE ''%promote%'' OR ' **+** **@**columnName **+** ' LIKE ''%champion%'' OR ' **+** **@**columnName **+** ' LIKE ''%endorse%'' OR ' **+** **@**columnName **+** ' LIKE ''%commend%'' OR ' **+** **@**columnName **+** ' LIKE ''%propose%'' OR ' **+** **@**columnName **+** ' LIKE ''%oppose%'' OR ' **+** **@**columnName **+** ' LIKE ''%condemn%'' OR ' **+** **@**columnName **+** ' LIKE ''%reject%'' OR ' **+** **@**columnName **+** ' LIKE ''%disapprove%'''; **SET @sql =** ' SELECT * INTO [SORecommendationsDb].[dbo].[Comments_Like] FROM [StackOverflowCommentsDb_Oct2025].[dbo].[StackOverflowComments] WHERE ' **+ @**likeKeywords **+** '; '; **EXEC** sp_executesql **@sql**;

**Box 16:** Extracting LIKE - CONTAIN records which may have false positives / negative results or can be still useful in some aspect.

**Questions Title**
SELECT * into [SORecommendationsDb].[dbo].[QuestionsTitle_LikeMinusContain] FROM [SORecommendationsDb].[dbo].[QuestionsTitle_Like] l WHERE NOT EXISTS ( SELECT 1 FROM [SORecommendationsDb].[dbo].[QuestionsTitle_Contain] c WHERE c.AutoIdPK = l.AutoIdPK );
**Questions Body**
SELECT * into [SORecommendationsDb].[dbo].[QuestionsBody_LikeMinusContain] FROM [SORecommendationsDb].[dbo].[QuestionsBody_Like] l WHERE NOT EXISTS ( SELECT 1 FROM [SORecommendationsDb].[dbo].[QuestionsBody_Contain] c WHERE c.AutoIdPK = l.AutoIdPK );
**Answers**
SELECT * into [SORecommendationsDb].[dbo].[Answers_LikeMinusContain] FROM [SORecommendationsDb].[dbo].[Answers_Like] l WHERE NOT EXISTS ( SELECT 1 FROM [SORecommendationsDb].[dbo].[Answers_Contain] c WHERE c.AutoIdPK = l.AutoIdPK );
**Comments**
SELECT * into [SORecommendationsDb].[dbo].[Comments_LikeMinusContain] FROM [SORecommendationsDb].[dbo].[Comments_Like] l WHERE NOT EXISTS ( SELECT 1 FROM [SORecommendationsDb].[dbo].[Comments_Contain] c WHERE c.AutoIdPK = l.AutoIdPK );

**Table 7:** Results of Keywords Based Filtering for Software Recommendations Related Insights Present in Stack Overflow Data Dump (October 2025).

Data	Total Records	Substring Matching (LIKE)	Exact Matching (CONTAINS)⁎	Substrings Other than Exact Match (LIKE-CONTAINS)⁎
**Question Title**	24,198,178	164,586 (0.68%)	73,901 (0.31%)	90,685 (0.37%)
**Question Body**		2646,945 (10.94%)	1135,148 (4.69%)	1511,800 (6.25%)
**Answers**	36,063,114	3655,886 (10.14%)	2228,118 (6.18%)	1427,770 (3.96%)
**Comments**	91,315,031	4014,709 (4.40%)	1900,242 (2.08%)	2114,468 (2.32%)

⁎ Included in the dataset published in the corresponding data repository.

### 4.4 Additional metadata extraction

The data mentioned in Table 7 forms the core part of SORD. To enrich the data and effectively utilize it for training purposes, we extracted the *Users* who posted such insights, the *Badges* attained by those users and the *Votes* assigned to the extracted posts so that those who may wish to utilize SORD do not need to preprocess the gigantic Stack Overflow data dump again for their extraction. Similarly, we also considered all the posts directly or indirectly referred to in the SORD (directly as PostId in Questions and Comments or indirectly as ParentId in Answers) and considered their associated Tags. We then extracted the unique tags with their frequency of occurrence in SORD directly or indirectly (directly in case of Questions that have the keywords in Title or Body or indirectly in case of questions associated with filtered Answers and Comments). Table 8 provides the results.

**Table 8:** Additional Metadata Extracted from Stack Overflow Data Dump for Enriching SORD.

Data	Total Records	Filtered Records Related to SORD
**Badges**	54,312,436	34,830,450 (64.13%)
**Tags**	66,046	61,672 (93.38%)
**Users**	30,088,388	1993,211 (6.62%)
**Votes**	246,514,372	50,725,814 (20.58%)

### 4.5 Exporting SORD to CSV

The dataset is provided in CSV format and was exported directly from Microsoft SQL Server Management Studio using the “Save Results As” functionality applied to standard SELECT * queries. The exported files use a comma (,) as the field delimiter and include a header row corresponding to column names. Missing values are represented as empty fields, consistent with default SQL Server export behavior. The underlying SQL Server tables contain text fields that may include HTML content, and such content has been retained without modification during export; no HTML parsing, cleaning, or removal was performed. The CSV files were generated without additional post-processing, ensuring that they directly reflect the stored database content. Encoding follows the default system configuration of SQL Server Management Studio at the time of export.

### 4.6 System and software specifications

All the methodological steps were performed using a desktop machine with the hardware and software specifications given in Table 9.

**Table 9:** System Specifications and Software Versions.

System Specifications	
Operating System	Windows 11 Pro (24H2)
RAM	64 GB
Processor	Intel (R) Core TM i5–9600 CPU @ 3.10 GHz
Storage	5.5 TB HDD (SATA) HGST HUH726060ALE610, 239 GB SKHynix_HFS256GD9TNG-L5B0B
**Software Versions**	
MS SQL Server	Microsoft SQL Server 2022 Developer Edition (64-bit)
SQL Server Management Studio	20.2.1
Anaconda Distribution	Anaconda3 2025.06 (64-bit)
Python (installed through Anaconda)	3.13.5
Jupyter Notebook (installed through Anaconda)	7.3.2
Git (for using Git Bash)	Git-2.50.1-windows.1
7zip	Not required due to native support in Windows 11

Experiments were conducted on a workstation with 64 GB of RAM (described above), reflecting the hardware available for testing. However, this should not be interpreted as a minimum requirement. The method has not been systematically evaluated on lower-memory systems; thus, runtime and memory usage may vary depending on hardware configuration. Nevertheless, the scalable preprocessing approach employed in this work is expected to allow the method to run on systems with less memory, with only minor adjustments to processing parameters such as number of lines to split large XML files. Further details for readers with memory and or storage constrained environments are available in the related article [2].

### 4.7 Preprocessing and extraction time

The time (measured from a single experimental run) taken to preprocess the Stack Overflow data dump published in October 2025 on the above-mentioned machine is given in Table 10. It is intended to provide a general reference for the readers and should be interpreted as an approximate estimate rather than an exact benchmark. Variations may occur depending on hardware and system load. The following three steps were considered for time calculations.

• Step 1 (S_1_): Converting large sized XML files to smaller chunks using Git Bash (split-by-line)
• Step 2 (S_2_): Converting generated chunks to valid XML files
• Step 3 (S_3_): Reading small XML files and saving data to MS SQL Server

**Table 10:** Time Taken to Preprocess Stack Overflow Data Dump Published in October 2025.

File Name	Total Chunks	Time Taken (seconds)						
		S_1_		S_2_		S_3_		Total Time /File (TS_1_ + TS_2_ + TS_3_)
		Total (TS_1_)	Avg. / Chunk	Total (TS_2_)	Avg. / Chunk	Total (TS_3_)	Avg. / Chunk	
Badges	1087	86.96	0.08	130.44	0.12	6880.71	6.33	7098.11 (01:58:18)
Comments	1827	347.13	0.19	529.83	0.29	13,665.96	7.48	14,542.92 (04:02:23)
PostHistory	3247	2207.96	0.68	4156.16	1.28	107,118.5	32.99	113,482.7 (31:31:23)
PostLinks			-		-	817.71	-	817.71 (00:13:38)
Posts	1208	1220.08	1.01	1775.76	1.47	42,171.28	34.91	45,167.12 (12:32:47)
Tags			-		-	8.75	-	8.75 (00:00:09)
Users	602	90.3	0.15	138.46	0.23	8584.52	14.26	8813.28 (02:26:53)
Votes	4931	345.17	0.07	493.1	0.1	7495.12	1.52	8333.39 (02:18:53)
**Total Time / Step (hr:min:*sec*)**		**4297.6** **(01:11:38)**		**7223.75** **(02:00:24)**		**186,742.6** **(51:52:23)**		**198,263.9** **(55:04:24)**

After successfully saving the data present in the data dump to MS SQL Server database tables, we extracted *Questions* and *Answers* from *Posts*. It took 02:37:28 to extract *Questions* and 01:52:25 to extract *Answers* from *Posts* using the machine described in Table 9. Once the *Questions, Answers* and *Comments* were present in separate MS SQL Server database tables, we proceeded with actual recommendation related keyword-based data extraction process followed by extraction of additional metadata. The file sizes of the extracted dataset along with time taken for extraction are given in Table 11. Cumulatively, it took us >68 h (2.88 days) to curate SORD from the Stack Overflow data dump published in October 2025.

**Table 11:** File Sizes and Extraction Time of Data Included in SORD.

Folder	File Name	File Size	Extraction Time (hr:min:*sec*)
questions	QuestionsTitle_Contain.csv	118 MB	00:03:58
	QuestionsBody_Contain.csv	3.26 GB	00:47:31
	QuestionsTitle_LikeMinusContain.csv	178 MB	00:00:32
	QuestionsBody_LikeMinusContain.csv	3.89 GB	00:14:20
answers	Answers_Contain.csv	3.27 GB	00:43:04
	Answers_LikeMinusContain.csv	2.10 GB	00:07:17
comments	Comments_Contain.csv	586 MB	00:04:57
	Comments_LikeMinusContain.csv	634 MB	00:01:39
additional-metadata	FilteredBadges.csv	2.15 GB	00:02:25
	FilteredTags.csv	982 KB	00:00:29
	FilteredUsers.csv	305 MB	00:02:16
	FilteredVotes.csv	2.77 GB	00:05:54
Intermediate database tables not included in SORD	QuestionsTitle_Like	-	00:21:35
	QuestionsBody_Like		02:20:35
	Answers_Like		01:47:42
	Comments_Like		00:52:23
	FilteredPosts		01:57:07
Total		19.22 GB	09:33:44

### 4.8 Keyword-wise distribution of data included in SORD

The number of records retrieved for each keyword across each of the 8 different csv files corresponding to questions, answers and comments is presented in Table 12. A substantial variation in record counts is observed, with values ranging from near-zero entries in case of some keywords to several hundred thousand in others. For instance, certain keywords such as advocate, endorse, praise, champion, condemn and disapprove yielded fewer than a few hundred records across all sources, whereas others consistently returned counts exceeding thousands in multiple categories. This wide range indicates a highly uneven distribution, where a subset of keywords contributes disproportionately to the overall dataset, while others have a comparatively minor impact. Such variability reflects the sensitivity of the data retrieval process to keyword selection, as differences in keyword specificity, relevance, and prevalence across sources directly influence the volume of retrieved records. The selected keywords were identified based on their meanings and relationships as defined in standard English dictionaries, ensuring general semantic coverage. However, despite being synonyms or antonyms of recommend, not all keywords may equally be prevalent across specific domains (e.g., software engineering as compared to medical, or other specialized fields), which may contribute to the observed variability. Nevertheless, the inclusion of a diverse set of keywords helps to broaden dataset coverage and reduce reliance on any single term. It should be noted that alternative keyword choices, particularly those tailored to domain-specific usage, may lead to differences in dataset composition and overall record distribution, and thus warrant further investigation in future work.

**Table 12:** Keyword-wise Distribution of Data Included in SORD (*C = Contain, L-C = LikeMinusContain).

Sr.	Keyword	Questions Title		Answers		Answers		Comments		Total
		C	L-C	C	L-C	C	L-C	C	L-C	
1	Recommend	1383	9438	65,613	136,130	554,700	209,656	349,984	207,607	**1534,511**
	Synonym									
2	Advise	776	268	85,363	15,302	56,517	19,286	68,151	20,928	**266,591**
3	Advocate	4	4	827	598	3873	1026	4638	1205	**12,175**
4	Adopt	249	533	7301	12,614	16,305	17,134	9936	13,119	**77,191**
5	Suggest	3164	14,411	326,631	776,025	647,799	481,424	644,795	1185,428	**4079,677**
6	Suitable	5511	99	59,805	3042	83,794	2148	75,598	2835	**232,832**
7	Praise	12	4	602	651	617	427	1061	475	**3849**
8	Favor	306	1675	12,122	36,377	27,891	42,839	25,857	27,413	**174,480**
9	Support	59,423	47,960	620,148	414,747	967,450	526,648	723,718	468,595	**3828,689**
10	Urge	22	4025	4036	41,975	7552	30,716	4973	31,689	**124,988**
11	Promote	496	407	5485	5522	9841	10,551	7681	8599	**48,582**
12	Champion	20	35	1676	2373	1268	1578	1254	1859	**10,063**
13	Endorse	17	254	1071	3468	1343	2279	1101	1742	**11,275**
14	Commend	1465	9471	66,901	136,829	555,881	210,768	352,476	209,035	**1542,826**
15	Propose	96	239	9540	24,478	31,145	43,286	27,983	63,792	**200,559**
	Antonym									
16	Oppose	43	1386	3327	33,236	8505	48,628	3386	57,565	**156,076**
17	Condemn	0	2	88	153	101	154	167	288	**953**
18	Reject	2564	10,111	57,482	67,190	64,697	38,318	28,214	45,218	**313,794**
19	Disapprove	9	14	298	273	260	176	303	176	**1509**
	**Total**	**75,560**	**100,336**	**1328,316**	**1710,983**	**3039,539**	**1687,042**	**2331,276**	**2347,568**	**12,620,620**

### 4.9 Getting started with the SORD

To reduce the barrier for novice users, in this section we provide guidance for loading and examining the SORD. The dataset originates from XML based data dump stored in a set of primary tables (mentioned in Table 6). Some columns in these primary tables were found to provide no additional information for any record in the published dataset and were therefore excluded; the list of removed columns is documented in the replication package (\code\Generating_SORD.ipynb). Filtered subsets were then generated using SELECT INTO queries, resulting in derived tables (e.g., QuestionsTitle_Contain, QuestionsTitle_LikeMinusContain and so on). As these derived tables are fully determined by the primary tables and transformation queries, explicit CREATE TABLE statements for the derived tables are not included; instead, providing the schemas of the primary tables (excluding the omitted columns) along with the associated queries is sufficient to reproduce all intermediate and final datasets.

For practical use, the dataset is also provided in CSV format and can be directly imported into Microsoft SQL Server for inspection and analysis. Notably, SQL Server Management Studio allows CSV import without requiring pre-created tables, enabling quick setup for users [28]. After loading, users can easily examine a small subset of the data using simple preview queries (e.g., SELECT TOP 10 * FROM table_name), facilitating quick verification and exploration.

## 5 Portability across other relational database systems

The data curation procedure presented in this paper was implemented using MS SQL Server and is an extension of an end-to-end preprocessing pipeline designed for scalable conversion of continuously growing XML based data dumps of Stack Exchange Network based Q&A sites, particularly Stack Overflow, comprising of millions of textual records [2]. This core pipeline involves multiple stages: (i) chunking large-sized single XML files to multiple smaller chunks (using gitbash), (ii) converting smaller chunks to valid XML files (using python scripts), (iii) creation of database(s) and tables corresponding to XML files included in the data dump (using SQL scripts), (iv) storing data from small XML files to SQL Server tables (using python scripts utilizing pandas and pyodbc), (v) construction of indexes (including full-text indexes which is only possible with customized SQL Server installation by enabling full-text search feature). The extended pipeline (used for curation of SORD once the XML based data dump has been converted to SQL Server database records) involves (vi) execution of keywords-based filtering using pattern-matching (LIKE) and full-text search (CONTAINS) predicates.

Reproducing the complete pipeline in alternate database environments would require systematic adaptation of XML parsing mechanisms, database version selection, schema design, indexing configurations and full-text search setup. A full reimplementation of this end-to-end workflow is beyond the scope of this work. However, for those interested in reproducing the steps on relational databases other than MS SQL Server, this section intends to provide a general guideline highlighting the key points for consideration.

A key challenge in the above-mentioned workflow arises from the continuously growing quarterly updated XML based Stack Overflow data dump which, over the period, has become too large to be efficiently processed in a single run using conventional in-memory parsing approaches. To address this limitation and ensure scalability, gitbash’s split command followed by pandas was employed as an intermediate processing layer to enable chunk-wise ingestion of the XML based data into tabular form. Specifically, the XML file was partitioned into smaller segments, which were sequentially parsed into data frames and subsequently inserted into SQL Server tables using pyodbc with batch insertion (executemany). This design enables memory-efficient processing while maintaining compatibility with relational storage operations and provides extensibility for future dataset growth, as the data volume is expected to increase over time.

Even in case of SQL Server, the Express Edition limits the database size to 10GB [29]. Hence, processing the data dump using express edition was not possible (see Table 3 for data dump file sizes). We, therefore, utilized SQL Server Developer Edition which does not impose any limit on database size. However, it can’t be used in production environments. Hence, interested readers must evaluate such limitations in case of alternate database management systems.

Reading data from smaller XML chunks to pandas dataframes and then saving the data into SQL Server tables is next point for consideration. Readers may need to update the relevant python scripts based on their chosen database, particularly database drivers. Although the executemany method used for bulk insertion of data to SQL Server is not SQL Server specific, but it may not behave consistently in case of other databases. Furthermore, the insertion performance may significantly vary across databases as the pyodbc has a special optimization for SQL Server i.e. cursor.fast_exceutemany=True which massively speeds up bulk inserts. This may not work for MySQL or PostgreSQL ODBC drivers. Additionally, changing libraries for insertion or database drivers will require changing syntax for SQL queries. This is equally true for database and table creation queries including the data types used to store data and creation of corresponding indexes as well.

SQL Server was preferred as the Stack Exchange Network itself uses SQL Server [30] to store data of its 182 Q&A sites so data quality was ensured by following the data types used by Stack Exchange Network. The choice of SQL Server was guided by practical considerations associated with large-scale text processing, including native support for XML handling, integrated full-text indexing capabilities, and mature query optimization mechanisms for text-intensive workloads. While comparable functionality exists in other relational database management systems such as MySQL and PostgreSQL, their configuration requirements, indexing strategies, and text search implementations differ.

Although this study does not assert inherent superiority of a specific database system and reflects an implementation aligned with the requirements of the dataset and processing pipelines, previous studies involving benchmarking experiments with datasets of up to several million records demonstrated that SQL Server provides better scalability, performance and query optimization for larger workloads [31,32].

The final query layer responsible for extracting recommendation related questions, answers and comments used pattern matching (through LIKE) and full-text search (through CONTAINS) operators. The LIKE-based filtering strategy is largely portable across relational database systems, as it relies on standard SQL syntax. Queries of the form:

WHERE columnname LIKE '%keyword%' can be directly translated across platforms, with minor adjustments required primarily for case sensitivity handling (e.g., the use of ILIKE in PostgreSQL or collation-dependent behavior in SQL Server and MySQL). In contrast, full-text search functionality is inherently vendor-specific. The SQL Server predicate:

WHERE CONTAINS (columnname, '``keyword1” OR ``keyword2'' OR …') can be approximated in MySQL using:

WHERE MATCH (columnname) AGAINST ('keyword1 keyword2 …' IN BOOLEAN MODE) and in PostgreSQL using:

WHERE to_tsvector ('english', columnname) @@ to_tsquery ('keyword1 | keyword2 | …')

Although these formulations provide functionally similar keyword-based retrieval, full-text search implementations differ significantly across systems with respect to tokenization, stopword removal, stemming, and indexing strategies. These differences become increasingly relevant at scale where variations in indexing efficiency and query execution strategies can influence performance resultantly causing variations in both query execution behaviour and the exact set of retrieved records. Consequently, even when equivalent queries are employed, the exact set of retrieved records may not be identical across platforms. Therefore, while the logical structure of the filtering queries can be preserved, exact reproducibility of results across platforms is not guaranteed.

In summary, while the logical structure of the filtering queries can be adapted to other relational database systems, strict reproducibility requires alignment across preprocessing, indexing, and text search configurations. The use of SQL Server in this study reflects a practical design decision for handling large-scale XML-derived textual data, rather than a constraint on the general applicability of the proposed filtering approach.

Researchers seeking to replicate or extend this work in alternative environments are therefore encouraged to consider the above-mentioned system-level differences when implementing the described filtering strategy. Those seeking strict cross-system reproducibility may consider relying on LIKE-based filtering, acknowledging the associated trade-offs in linguistic sensitivity and computational efficiency.

## 6 Text preprocessing and data release considerations

The dataset introduced in this work represents a large-scale, heterogeneous collection of recommendation-oriented content extracted from Stack Overflow, spanning programming languages, tools, frameworks, technologies, and associated code artifacts. To the best of our knowledge, this dataset is unique in its scope and structure, as no comparable publicly available dataset exists with similar coverage of mixed natural language and code-based recommendation signals.

It is important to note that the dataset is intentionally released in a raw form. While this implies that additional processing is required before it can be directly used for specific downstream tasks, the primary motivation is to maximize its utility across a broad range of potential applications. Given the diversity of possible use cases, it is neither feasible nor desirable to enforce a single preprocessing pipeline at the time of release, as such choices would implicitly bias the dataset toward specific tasks while limiting its applicability for others. Instead, we anticipate that different research communities will apply domain-specific processing tailored to their particular objectives.

This design choice is also motivated by the aim of enabling unforeseen or emergent use cases. By preserving the original structure and content of the data, we allow future users to explore directions that may not have been anticipated during dataset construction, including tasks that require syntactic, lexical, or structural information that would otherwise be removed by aggressive preprocessing.

Consequently, standard NLP preprocessing techniques (e.g., stopword removal, stemming, case folding, and punctuation filtering) were not applied prior to release. This decision is further motivated by the need to preserve data fidelity in a domain where textual and syntactic elements carry intrinsic semantic meaning. In software engineering discourse, tokens such as file extensions (e.g., “.py”, “.js”, “.exe”), versioned identifiers (e.g., “log4j”), and language-specific symbols (e.g., “C#”, “C++”) encode critical information that may be lost or distorted under conventional preprocessing pipelines.

Similarly, code snippets embedded in the dataset often reflect recommended programming practices and structural patterns. Standard NLP transformations may alter or remove syntactic cues that are essential for interpreting such recommendations. Preliminary exploratory analysis further indicated that the effects of preprocessing are highly heterogeneous across data types, reinforcing the need to preserve the original form.

Finally, releasing the dataset in its raw form is also intended to make inherent challenges in processing real-world software engineering text explicitly visible. An upcoming companion study will further analyze these challenges and provide structured guidance on preprocessing strategies suitable for different downstream tasks.

## 7 Analysis of insight stimulating example

Realizing the error-prone nature of keywords-based filtering which can include false positives, we considered a small subset of sample records to answer the following research question.

• RQ3: Do Stack Overflow discussions containing recommendation related keywords contain implicit reviews that can be converted to useful recommendations?

To answer this RQ, we tried to find answers to a question asked on Software Recommendations Stack Exchange site through Comments_Contain included in SORD. Out of QuestionsTitle, QuestionsBody, Answers and Comments, we considered Comments as a representative sample (being shorter in length and providing precise information without additional information/noise to be removed) for anaylsing the results. To better contextualize the readers about the usefulness of SORD extracted using keywords-based approach, we considered a popular question from Software Recommendations site and tried to compare the answers posted on Software Recommendations to relevant Stack Overflow comments extracted from Comments_Contain. The results proved to be rather promising.

### 7.1 Sample question selection

As questions asked on Software Recommendations can be asked by end users of software as well as software developers, to select a sample question particularly relevant to software developers we considered the top 5 development related tags out of top 20 tags on Software Recommendations site (as included in the data dump of Software Recommendations site published in October 2025 data dump). Table 13 enlists the top 20 tags on Software Recommendations site with the top 5 development related tags highlighted.

**Table 13:** Top 20 Tags on Software Recommendations Stack Exchange Site.

No.	Tag Name	Tag Count	No.	Tag Name	Tag Count
1	windows	3812	11	java	702
2	Gratis	2309	12	database	643
3	linux	2152	13	software-development	642
4	android	2017	14	video	598
5	open-source	1813	15	c++	572
6	web-apps	1306	16	osx	571
7	library	1029	17	audio	507
8	python	873	18	web-development	496
9	javascript	840	19	file-management	493
10	pdf	711	20	html	480

As a representative sample for analysis, we considered the top 5 questions asked on Software Recommendations site having an answer accepted by the questioner and having one or more of the top 5 development related tags highlighted in Table 13. The corresponding top 5 questions from Software Recommendations site are given in Table 14.

**Table 14:** Top 5 Software Development Related Questions Asked on Software Recommendations Site.

Id	Creation Date	Score	Views	Title (and Tags)	Ans.	Comments	Cumulative Ans. Score
32,612	2016–06–03	35	302,491	GUI drag and drop style GUI Builder for Python Tkinter *windows, open-source, software-development,****python****, gui-builder*	6	0	42
13,856	2014–11–12	11	98,378	Is there an app that runs Java (.jar) files on Android? android, java, emulator	5	0	12
14,797	2014–12–22	12	64,681	Software to convert HTML, CSS and JavaScript into an exe? windows, javascript, html, css	4	1	17
7463	2014–07–02	**29**	59,030	Fastest free Python library to read a CSV file with 1–3 columns of numbers? *gratis,****library****,****python****, csv, performance*	**6**	**2**	**48**
17,812	2015–03–10	19	49,418	Java library that diffs JSON and generates what was added/deleted/modified?	2	1	24
				***library*, java,***json*			

Out of the top 5 questions we considered two (first and fourth) questions for further analysis with Id 32,612 and 7463. Although the first question had higher score than the other one and had equal number of answers, the fourth one had greater cumulative answer score and more comments. Hence, we selected the fourth question (Id=7463) for further analysis. We preferred the fourth question for having a higher cumulative answer score because it reflects stronger overall validation and engagement from the community. When multiple answers collectively receive a high number of upvotes, it indicates that the question has attracted several useful, well-regarded solutions rather than relying on a single response. This not only increases confidence in the correctness of the information but also provides a richer set of perspectives, approaches, and edge-case considerations. Additionally, such questions are often more relevant or commonly encountered, making them more valuable for a broader audience. In this way, cumulative answer score serves as a practical proxy for both the quality and usefulness of the discussion surrounding a question. Detailed attributes associated with the chosen question are given in Table 15 along with a summary of answers provided by users on Software Recommendations site.

**Table 15:** Sample Software Development Related Question Asked on Software Recommendations Site.

Question (Title)	Fastest free *Python library* to *read* a *CSV* file with 1∼3 columns of numbers
Question (Body)	I am looking for the fastest Python library to read a CSV file (if that matters, 1 or 3 columns, all integers or floats, example) into a Python array (or some object that I can access in a similar fashion, with a similar access time). It should be free, work on Windows 7 and Ubuntu 12.04, and with Python 2.7 × 64.
Tags	gratis, library, python, csv, performance
Creation Date	2014–07–02
Last Edit Date	2020–09–14
Last Activity Date	2021–11–08
Accepted Answer ID	7510 (accepted on 2014–07–03)
Recommendations (Answers)	pandas.io.parsers.read_csv (accepted answer, recommended in 2014) Numpy's from_file (recommended) and loadtext (slow not recommended)(2014) fastcsv (recommended in 2016) Python package for data mining: DaPy (recommended in 2018) pydatatable package (recommended in 2018) Standard Python csv library (recommended in 2018 and 2023) (overall most recommended)

Although various options were provided in answers and comments, the primary competition was between *pandas* and Python's built-in *csv module*. Numpy was overall not recommended whereas *fastcsv* was a somewhat better option at some point in time, but it got deprecated and no longer remain a good option. The answer suggesting *pydatatable* received only 1 vote whereas *DaPy* received no vote at all depicting low quality recommendations as compared to remaining 4 answers getting 34, 9, 6 and 1 vote respectively. Hence, we expected to get *pandas* and Python's built-in *csv module* as common recommendations in response to this question.

### 7.2 Analyzing the presence of implicit/explicit reviews/ recommendations in SORD

To filter potentially relevant comments from SORD, we considered *subject, object* and *verb* from the sample question (see question title in Table 15) as keywords to represent the information need of questioner. We used *python, library, csv* and *read* as keywords and tried to find relevant comments from the Comments table representing original Stack Overflow data dump and Comments_Contain and Comments_LikeMinusContain included in SORD. We found 1068 comments from the Comments table in Stack Overflow data dump, which are obviously not feasible for an average procrastinate user to read. On the other hand, the same set of keywords when applied to our extracted dataset of recommendation related Comments i.e. Comments_Contain and Comments_LikeMinusComments returned only 60 and 24 comments respectively. The 60 relevant comments from Comments_Contain are given in Table 16.

**Table 16:** Recommendations Related Comments from SORD Comments_Contain Related to Python Library for Reading CSV Files.

Id	Year	Score	Text
9310,050	2011	0	+1, but suggest `newline=''` instead of `r` per Python 3 [csv.reader] (http://docs.python.org/py3k/library/csv.html?highlight=csv#csv.reader) docs.
10,184,850	2011	0	@mantissa45: If you use [csv.reader()`](http://docs.python.org/library/csv.html\#csv.reader), then your row is probably suitable for the first case I mentioned in my previous comment - `'{0} {1}'.format(*row)` - and yes, the number within curly brackets is the index of the column (starting with `0` for the 1st column).
12,305,083	2012	0	No, SSIS is package-based so you can't avoid creating one. Personally, if I couldn't use SSIS then I would use a language that already has CSV library support (like Perl or Python) because it's harder than it first seems to write CSV files. Your code doesn't appear to handle the case where a string contains quotes, for example.
16,857,084	2012	0	The `dialect` parameter to `csv.reader` describes how rows and columns are delimited in your CSV file. If you know the format in advance, you can skip detecting it with `csv.Sniffer` (which is not always going to get it right) and simply provide the correct delimiters instead. I suggest reading [the `csv` module's documentation] (http://docs.python.org/library/csv.html) for more details.
19,557,973	2013	0	If you are just trying to persist a dictionary on file (you seem to be writing it and reading it back again), I would not suggest using CSV. Instead look into just serializing and writing out the dictionary to a file, python has a very nice module called Shelve, which automates this process nicely: http://docs.python.org/2/library/shelve.html
28,437,060	2013	0	I recommend using `csv.dictreader` to turn your csv into a dictionary and then just accessing the values for column 11 and column 38 by their fieldnames. (see http://docs.python.org/2/library/csv.html and various examples at SO).
31,668,541	2014	0	You don't need to use a path. Wrap the result from the http request in a StringIO instance, and then pass that to the csv reader. You should read the docs more carefully for csv reader. http://docs.python.org/2/library/csv.html - it says - Return a reader object which will iterate over lines in the given csvfile. csvfile can be any object which supports the iterator protocol and returns a string each time its next() method is called - file objects and list objects are both suitable.
33,795,418	2014	0	I would highly recommend looking into the [`cvs`] (http://docs.python.org/2/library/csv.html) module. That will let you write out without manually stitching your rows together and when you read it back in each row will be a list
35,488,006	2014	0	I would suggest reading the data with the [Python CSV library] (https://docs.python.org/2/library/csv.html).
35,530,442	2014	1	There are numerous issues with your code, which I understand is because you're very new to Python. I strongly recommend investing the time to read through the [tutorial] (https://docs.python.org/2/tutorial/) (or [this] (https://docs.python.org/3/tutorial/) if you're using Python 3.x). Also study the [`csv` module docs] (https://docs.python.org/2/library/csv.html), which include some examples how to use it.
36,160,617	2014	0	@mgilson not sure if I've got you right. Actually, [csv.reader] (https://docs.python.org/2/library/csv.html\#csv.reader) ***doesn't seem to be supposed to accept filenames***. It accepts any iterable that returns strings which will go horribly wrong on a filename. As 'streams', it definitely also accepts not just file-like objects though, but any other suitable iterable objects, if that's what you meant.
38,249,640	2014	0	Are you writing your custom [item exporter] (http://doc.scrapy.org/en/latest/topics/exporters.html\#module-scrapy.contrib.exporter)? Scrapy has built-in support for CSV output. But if you want to customize it, you could probably subclass [`CsvItemExporter`] (https://github.com/scrapy/scrapy/blob/master/scrapy/contrib/exporter/__init__.py\#L163). And read what Python's [csv] (https://docs.python.org/2/library/csv.html) has to say about Unicode input (that input should already be UTF-8 preferably).
38,804,331	2014	0	This [site] (http://www.python-excel.org/) should be useful. But I recommend you work with .csv files. It is much easier to manipulate data in csv using python ([read here] (https://docs.python.org/2/library/csv.html)). You can always save your xlsx files as csv and vice-versa. Read [this](http://stackoverflow.com/questions/3207219/how-to-list-all-files-of-a-directory-in-python) on how to access files of a folders in python
40,857,746	2014	0	I believe there are more easier and efficient ways to perform this outside Tableau. If you intend to use tde extract API, I assume you have some knowledge in some programming language. If you know python, I recommend you learning pandas library (if you don`t know it already). With that you can easily handle those multiple csv files and create a new one (or even export to MySQL, for instance, what would be smarter).
40,988,272	2014	0	Read about [CSV encoding] (https://docs.python.org/2/library/csv.html) support. There is an example UnicodeWriter right there in the CSV module reference doc.
47,124,139	2015	0	then i'd suggest provide your own reader to skip the bad bytes. open the file with `f = codecs.open("whatever.csv", error='ignore'); pandas.read_csv(f, delimiter=";")` https://docs.python.org/2/library/codecs.html
47,458,734	2015	0	Note that your current attempt puts *the string `worddict`*, not the dictionary you just carefully built, into `puzzle`. I suggest you read https://docs.python.org/2/tutorial/datastructures.html\#dictionaries and https://docs.python.org/2/library/csv.html - `csv.DictReader` can do most of this for you.
47,877,474	2015	1	The [examples] (https://docs.python.org/2/library/csv.html\#examples) section in the csv documentation says ``The csv module doesn't directly support reading and writing Unicode, but it is 8-bit-clean save for some problems with ASCII NUL characters. So you can write functions or classes that handle the encoding and decoding for you as long as you avoid encodings like UTF-16 that use NULs. UTF-8 is recommended.'' Note this is only part of your problem. The other is trying to read the JSON file line-by-line.
49,544,254	2015	1	I think, using pandas IO tools (http://pandas.pydata.org/pandas-docs/stable/io.html) for just reading and writing csv-files is kind of taking a sledgehammer to crack a nut. Nevermind, if you have pandas already installed on your system, but keep in mind that pandas is a relatively large python package. I'd rather suggest using `str.split` (https://docs.python.org/3.4/library/stdtypes.html\#str.split) or python's standard csv module (https://docs.python.org/3.3/library/csv.html)
50,157,418	2015	0	Since SO is not a code-writing platform, you should first try something yourself. You can do what you need using Python. As a starting point I would suggest to read something about file-handling (https://docs.python.org/3.4/tutorial/inputoutput.html\#reading-and-writing-files), [csv-files] (https://docs.python.org/3.4/library/csv.html) and strings (https://docs.python.org/3.4/library/stdtypes.html\#text-sequence-type-str).
51,303,044	2015	0	Possibly, using Win32com. It's horrible and poorly documented. I would recommend [xlrd] (https://pypi.python.org/pypi/xlrd) for reading older (non `.xlsx`) formats. Or more simply still, I notice the file you have hard-coded above is a `.csv` file. In which case look at the standard [csv] (https://docs.python.org/2/library/csv.html) library.
56,031,937	2015	1	To quote the ``csv'' factoid from http://wooledge.org/∼greybot/meta/csv: A csv file contains ``Comma Separated Values''. It represents records as lines and fields delimited by commas (though the delimiter can vary). Very simple CSV files can be parsed using a `while IFS=, read -a fields` loop. For more complete support, see 〈http://to.lhunath.com/bashlib\#L550〉, 〈http://docs.python.org/library/csv.html〉 or the csvtool / csvkit commands.
57,723,036	2016	0	I would suggest using the csv reader module to do this work instead of splitting it manually yourself. https://docs.python.org/3/library/csv.html. Also look at the dictreader function https://docs.python.org/3/library/csv.html\#csv.DictReader
66,615,325	2016	0	You can do that in any language that can read files. Python has special library support for CSV parsing - just google it
67,102,211	2016	0	I'd suggest [`pandas`] (http://pandas.pydata.org/pandas-docs/stable/timeseries.html) - it's a library to do this kind of timeseries analysis using Python. You could use `ts = read_csv …` to load from CSV, `ts.resample('5min').mean()` to take an average of 5 min of results, then `ts.plot()` to visualise. Happy to demo if you share your data?
67,766,283	2016	1	If you haven't already, I recommend that you skim the official documentation for the `csv` module. Pay especial attention to the code examples there: https://docs.python.org/3/library/csv.html
68,626,585	2016	0	Searching for ranges is a little tricky, but FWIW, you can read your data in a more convenient way using the [csv] (https://docs.python.org/3/library/csv.html) module. I suggest reading your file into a list of dictionaries, one dictionary per book.
69,471,429	2016	0	Python 2.7′s `csv` reader doesn't support Unicode without some help. See the [examples] (https://docs.python.org/2/library/csv.html\#examples) at the bottom of the [csv documentation] (https://docs.python.org/2/library/csv.html). Better, switch to Python 3.
74,622,679	2017	0	Which value is ``matches won''? Suggest you read the file with the [`csv`] (https://docs.python.org/3/library/csv.html\#module-csv) module because that's the kind of file it is (and would be better than using a regular expression to parse the lines).
75,058,753	2017	0	Do you have any prototype of your code? Give more details of your files. What is the structure of these files? Are they tab or comma-delimited? If this is the case, I sugges that you read about the csv module (https://docs.python.org/3/library/csv.html).
86,573,029	2018	0	I'm not quite sure by what you mean by 'grab' but let's go with `parsing` to which i'd advise you to look into the [Python csv module] (https://docs.python.org/3/library/csv.html). If you do '`parse`' your data, i'd advise you do it at the start, and not each time you will need it, either you'll end up just reopening files from which you could have read earlier.
87,179,245	2018	0	Provide a sample of the .csv that reproduces the issue, and a [mcve] along with the full traceback of the error message. The stock `csv` module in Python 2.7 doesn't support Unicode so you read raw encoded data, and should be decoding it to Unicode, not encoding it. You're getting the `UnicodeDecodeError`, because you can't encode a byte string, so Python 2.7 is ``helpful'' and decodes it with the default ASCII codec, hence your error. Also see the [examples at the bottom of the csv documentation] (https://docs.python.org/2.7/library/csv.html?highlight=csv\#examples) for dealing with Unicode.
90,724,588	2018	0	I would strongly recommend using the **csv** module to perform read and write actions against CSV file types. You can find the documentation for this module [here] (https://docs.python.org/3/library/csv.html).
97,236,346	2019	0	If your `object` column is a custom class and not one of the built-in types, you will need to do some sort of serialization, like saving your object to `json`. Even pandas won't be able to save you from having to write something that will read the data from the file and know how to create your desired object from it. If you have control of your data before it gets into the csv format, I would recommend looking into [pickles] (https://docs.python.org/3/library/pickle.html). Dataframes in pandas also have a to/from pickle method.
97,536,331	2019	0	Yes, there are ways, and one would be to read the file and write out a new one (at the same time) without the rows/lines in it you don't want. Since this looks like a CSV file, I would suggest using the [`csv`] (https://docs.python.org/3/library/csv.html\#module-csv) module to do both.
97,537,233	2019	0	@hoyeung Nice! If you are interested in learning the `csv` module (or get stuck trying to deal with csvs in the future) I would recommend reading this page https://docs.python.org/3/library/csv.html there are other useful features like `csv.DictReader()` or specifying dialects and quotechars!
98,271,224	2019	1	Are you using the dataframe after doing the file renames? If not, I would recommend using the `csv` module instead, as [csv.reader](https://docs.python.org/3/library/csv.html\#csv.reader) may give you performance improvements and avoid reading from a separate data structure
98,644,087	2019	1	please post the python code you have written and point out where you ran into trouble… i suggest you use the [`csv` module] (https://docs.python.org/3/library/csv.html) (with `delimiter=":"`) to read the file.
101,454,764	2019	1	@pvarma If you see the read_csv documentation, it specifically says it accepts https, s3, e.t.c. But pickle documentation does not. And it does not support because pickle files are `not secure against erroneous or maliciously constructed data` as per their documentation (https://docs.python.org/3/library/pickle.html)
105,215,984	2019	0	CSV format is relatively easy to read and write, which would make ``extending'' it somehow to support nesting feasible to implement yourself - however doing so would make it non-conformant to the standard and likely not able to be read or written using Python's `csv` module in the standard library (as well as many other third-party) modules
106,538,185	2020	3	I'd suggest looking at [pd.read_csv] (https://pandas.pydata.org/pandas-docs/stable/reference/api/pandas.read_csv.html) or the [csv module] (https://docs.python.org/3/library/csv.html). It'd also help if you reduce the sample data in your example and provide what code you've tried and why it isn't producing the output you expect.
107,751,780	2020	2	R or Python are much more suitable to do analysis work on data sets. Your question is too broad. What exactly is it that you want to do? If you are asking how to parse CSV files, then you should look for a library. There is nothing by-default in C++ that does this for you. But library recommendations are off-topic here. For simple parsing, see [How can I read and parse CSV files in C++?](https://stackoverflow.com/questions/1120140/how-can-i-read-and-parse-csv-files-in-c).
108,052,474	2020	0	I suggest you use a csv.reader` (https://docs.python.org/3/library/csv.html\#csv.reader) to read the rows of data into your list.
109,244,875	2020	0	For example, did you try using the csv module (https://docs.python.org/3/library/csv.html) and use the datetime module (https://docs.python.org/3/library/datetime.html) to parse the dates? Someone (I'd almost put money on it) is about to suggest `pandas.read_csv` and it's not helpful in understanding anything
109,556,481	2020	1	Suggest do some reading about [reading and writing files] (https://realpython.com/read-write-files-python/), [common string operations] (https://docs.python.org/3/library/string.html), [csv files] (https://realpython.com/python-csv/) and maybe also [regular expressions] (https://docs.python.org/3/library/re.html)
110,134,854	2020	0	I'd suggest to read about the `csv` module. (https://docs.python.org/3.8/library/csv.html?highlight=csv\#module-csv) Parsing a csv file by yourself is almost never a good idea. Most selfwritten CSV parsers break when some funny strings occur.
111,711,659	2020	0	Yes - you'll need to pass a suitable CSV dialect object to the `csv.DictReader()` constructor (see https://docs.python.org/3/library/csv.html\#csv.Dialect). What does the CSV look like, what are the actual separators?
114,101,922	2020	0	I'm inclined to think it's something to do with either your data or the way you have read the data into the csv. But it's difficult to say without seeing at least part of your data. Have you used python's in-built [`csv.reader`] (https://docs.python.org/3/library/csv.html\#csv.reader) instead of [`pandas.read_csv`] (https://pandas.pydata.org/pandas-docs/stable/reference/api/pandas.read_csv.html)? Based on your error and based on this [answer] (https://stackoverflow.com/a/40726437/11542679) I would suggest trying with the pandas function.
114,766,535	2020	0	I'd recommend to use a [CSV reader] (https://docs.python.org/3/library/csv.html) for reading / parsing. With quoting this format can be more tricky than it looks like, and you don't need to reinvent the wheel.
119,000,798	2021	0	@DChase I'm not sure what you mean. Does each line already contain data that would make sense in a CSV? I suggest using [csv.writer] (https://docs.python.org/3/library/csv.html\#csv.writer)'s `writerow()` method, but I really need more information to make a meaningful recommendation. If you're having trouble using `csv.writer` and can't figure it out, feel free to post another question with details. I'll be glad to answer if you share the link here.
119,527,632	2021	1	As the name suggest, `DcitReder` is for reading. You need [`csv.DictWriter`] (https://docs.python.org/3/library/csv.html\#csv.DictWriter) for writing. Also, if working with pandas, look at `pandas.to_csv
120,909,126	2021	0	Using a IDE isn't going to help if you don't know the language or read the documentation. `for row CardIdentifier in csv\_file:` ***is*** syntactically incorrect. I suggest you review the documentation on the [`for`] (https://docs.python.org/3/reference/compound_stmts.html\#the-for-statement) statement as well on that of the [`csv.reader`] (https://docs.python.org/3/library/csv.html\#csv.reader).
124,657,547	2021	0	Well there is python support for basic csv operations in the `csv` package. Read more here https://docs.python.org/3/library/csv.html. Also look on the duplicate answer link
125,420,377	2022	0	While CSV files *are* text files, their contents is formatted in a [certain way] (https://datatracker.ietf.org/doc/html/rfc4180.html) - which you need to follow if you want it to be (or remain) valid. I strongly suggest you use the [`csv`] (https://docs.python.org/3/library/csv.html\#module-csv) module to read and write them.
126,353,549	2022	0	i suggest you use the `csv` module (https://docs.python.org/3/library/csv.html). [`DictReader`](https://docs.python.org/3/library/csv.html\#csv.DictReader) could simplify your code a lot!
132,354,038	2023	1	Look at the file in Notepad++ and what the line ending chars are-turn on ``hidden'' characters, or something like that. The lineterminator option, https://docs.python.org/2.7/library/csv.html, for the writer defaults to \'r\'n. Do you see that sequence, but doubled up, or do you see some other sequence? Maybe try fiddling with the option? Also, I think it's weird to be opening the output file for appending on every iteration of the reader. I'd recommend opening it once and creating the writer alongside the reader. Can 2.5 do multiple with-open statements? If not, just manually close the output
133,712,235	2023	0	An observation: a proper CSV file cannot have a \'n inside a value, unless it's within quotes. Especially if your CSV file has quotes, I would suggest to use an already existing library (e.g. https://docs.python.org/3/library/csv.html)
134,917,414	2023	0	Python has built in support for reading and writing CSV files; see https://docs.python.org/3/library/csv.html. You should use a `csv.writer`.
136,360,667	2023	0	I would suggest rewriting this to use [csv.DictReader] (https://docs.python.org/3/library/csv.html). Then other users can tell what you're doing. I've looked at this for a couple of minutes, and I have to keep flipping between CSV files and your code to try to figure out what it's doing.
139,477,480	2024	0	Please make an earnest attempt and provide an [mre] showing where you are stuck. If you really have no idea to start, I would suggest starting with searches for [How to Read and Write CSV files](https://stackoverflow.com/q/41585078/17030540), [Python csv library docs](https://docs.python.org/3/library/csv.html#module-csv), [How to rename a file in Python](https://stackoverflow.com/q/2491222/17030540), and the [Python 3 os.rename() docs](https://docs.python.org/3/library/os.html#os.rename)

In terms of search-space reduction, our approach reduced the results to 94.38%. On the other hand, in terms of coverage if we consider the two most valuable recommendations only i.e. *pandas* and *built-in csv module*, the results can be considered 100% [33]. On the other hand, if we consider all the 6 options discussed in answers/comments of the sample question (as mentioned in Table 15) with equal weightage, 33.3% items were covered by our keywords-based filtering.

The two competing options as identified by answers on *Software Recommendations* site, *pandas* and Python's built-in *csv module* both were found in the 60 filtered comments with their respective colors shown in Table 16. Without even reading the comments in detail, a reader can easily infer (through the highlighted text) that Python's built-in *csv module* is the all-time favorite option of developers being included in the default installation as compared to *pandas* (which was suggested in a smaller number of comments). On the other hand, several other software/technologies were mentioned in comments which were not directly relevant to the question, however, they may aid serendipitous discovery of knowledge e.g. *SSIS, Scrapy, Tableau* and so on. Furthermore, the *definition of CSV file format* was also found in the comments extracted. In addition to suggesting the use of Python's built-in *csv module*, these comments also provided links to learning resource /documentation for several other relevant Python constructs/datatypes/concepts e.g. *string, file handling, datetime module* and so on.

Interestingly, the original question asked on Software Recommendations site has been mentioned 9 times overall in the original Stack Overflow Comments out of which it appeared twice in Commnets_LikeMinusContain. These 2 comments were included in the 24 filtered comments for analysing the answers to this question. Another interesting insight that can be inferred from Table 16 is that *Python 2.x* has slowly been replaced by *Python 3* over the years. If we examine all the comments related to Python's built-in *csv module* containing links to official documentation, it is clear that from 2011 to 2017 both *2.x* and *3.x* were in use. However, from 2017 onwards the focus has been almost completely shifted to *3.x* whereby only 2 comments referred to documentation of *2.x* as compared to >25 comments referring *3.x*. This observation is aligned with rapidly evolving nature of Software Engineering data. As a *software, library, package* or *module* keeps on evolving (e.g. *Python 2.x* or *3.x*), their associated information spaces (e.g. *official documentation*) also evolve and produces more data, making information overload for developers more intense.

Reading the comments mentioned in Table 16, one can clearly understand that the Python's built-in *csv module* is the recommended option by most developers to deal with csv files. Hence, it can be stated that the comments containing recommendation related keywords contain implicit recommendations which can be converted to ratings.

Chronological analysis of answers mentioned in Table 16 clearly depicts that software engineering data (and the facts inferred from it) keep on evolving rapidly. Hence, a conclusive answer to such information needs may become outdated too quickly (e.g. *fastcsv* is the best option for reading csv files in Python). In such a scenario, presenting various options to the user and letting the user take the final decision based on the context is more appropriate which can be termed as *non-personalized recommendations.* For instance, in a comparison of option *x* with option y, while the questioner may have asked about the *fastest* option which seems to be *x*, the user may actually go for *y* because of the features offered by *y* or the lack of proper documentation, or lack of cross-platform support for *x* which may cause difficulties for the questioner (developer).

In order to evaluate our keywords based approach in terms of accuracy in identifying any sort of implicit or explicit developer preferences, reviews or recommendations, we utilized the 60 comments filtered from Comments_Contain (as given in Table 16) as a representative sample and highlighted both the true *positives* and *false positives*. The correctly identified comments vary in terms of intensity / polarity of opinions expressed e.g. *I recommend, I would suggest* and *I strongly recommended*. Some of the comments provided indirect recommendation by endorsing the opinion expressed in another answer or comment. One of the comments started with *+1* to endorse the answer to which the comment was associated. Furthermore, multiple comments had score greater than 0 which means that other users agreed with the suggestions provided / opinions expressed in such comments. These scores can be used as weights when inferring ratings from these comments.

An important observation regarding *false positives* is that most of them contained the keyword *support* which refers to the presence or absence of a particular feature e.g. *Python 2.7 doesn't support Unicode, the built-in support, the documentation recommends/suggests*. Although they may seem like negative results, such statements can provide an indication of features missing or present in a particular *package, library, module* (or any *software*). Hence, they can be utilized to infer ratings regarding any aspect of software under consideration to offer aspect-based recommendations [34] or multi-criteria recommendations [35].

We observed an interesting manifestation of the traits of software developers as knowledge workers [5] that although some of the comments strongly recommended / opposed something, they didn't actually explain the reasons behind such opinion e.g. *Pandas is worth it because it is so powerful, I'd almost put money on it, this doesn't need Pandas that is overkill, kind of taking a sledgehammer to crack a nut*. Such statements clearly indicate the presence of positive/negative polarity which can be found using sentiment analysis / opinion mining and then can be converted to ratings. However, accurately identifying polarity of Software Engineering based texts has its own associated challenges [36].

An even greater challenge is to correctly identify the entity about which the opinion has been expressed. This can be done at several different levels of granularity and can produce different results at each level. Identifying entities such as *python, pickle, pandas, built-in csv module* is one step and then identifying the complex and often hierarchical relationships among entities is another challenge e.g. *read_csv* is related to *pandas* whereas *csv.reader* is related to *built-in csv module*; both *pandas* and *built-in csv module* are related to *python* and *python* is related to *programming languages* and so on. Similarly, identifying the relationships between generic entities e.g. *python* with specific entities e.g. *python 2.x, python 3.x, python 2.7, python 3.5* is another challenge.

## 8 Estimating false positives in SORD using stratified sampling strategy

After demonstrating the utility of the dataset in a representative use case (in the previous section), in this section, we generalize the findings by assessing the dataset robustness at scale in terms of quantifying the rate and sources of noise introduced during keyword-based extraction. To complement the example analysis and assess the robustness of these observations at the dataset level and to quantify the rate and nature of false positives introduced by keyword-based dataset construction, we adopted a structured stratified sampling strategy that accounts for multiple sources of heterogeneity in the keyword set. The detailed steps followed are described below.

### 8.1 Dataset structure and sources of heterogeneity

The dataset was originally generated using a snowball sampling approach, where a primary seed keyword (recommend) was expanded into semantically related terms. This resulted in two broad groups of derived keywords: positively related terms or synonyms (14 keywords), which extend the semantic scope of the seed concept, and negatively related terms or antonyms (4 keywords), which capture semantically contrasting or indirectly associated contexts. In addition, prior analysis in Section 4.8 revealed strong heterogeneity in keyword frequencies. Furthermore, while manually analysing a few sample records for each keyword we observed distinct types of noise associated with specific terms, including gaming-related false positives (notably in case of champion, often appearing in variable-name contexts) and technical log-related ambiguity (notably in case of support, which appears in both true and spurious contexts).

Given this multi-dimensional structure, a purely frequency-based sampling strategy would risk under-representing critical semantic and error-prone regions of the keyword space. Therefore, we instead designed a stratified sampling approach along three axes: (i) **semantic role** (recommend, synonyms, and antonyms), (ii) **frequency distribution** (high-, mid-, and low-frequency keywords), and (iii) **known error propensity** (keywords identified as exhibiting systematic noise patterns).

Specifically, we selected a representative subset of keywords ensuring coverage of all three dimensions. This includes the seed keyword (*recommend*), multiple synonyms and antonyms spanning different frequency ranges, and explicit inclusion of potentially problematic cases (*support* for log-related ambiguity and *champion* for gaming-related false positives). Within each selected keyword, records were then randomly sampled from all dataset types (QuestionTitle, QuestionBody, Answers and Comments) and manually annotated to determine whether occurrences represented true positives or false positives. This design ensures that the resulting estimate reflects both the global false positive rate and the heterogeneous mechanisms contributing to noise across the dataset construction process.

### 8.2 Hierarchically stratified sampling design

#### 8.2.1 Definition of population strata

The dataset consists of 8 csv files (excluding additional metadata) containing recommendations related keywords extracted from Stack Overflow questions, answers and comments i.e. QuestionsTitle_Contain, QuestionsTitle_LikeMinusContain, QuestionsBody_Contain, QuestionsBody_LikeMinusContain, Answers_Contain, Answers_LikeMinusContain, Comments_Contain, Comments_LikeMinusContain respectively. Each of these files has different set of records corresponding to distinct keyword extraction method and data types. Hence, we treated each file as a separate population stratum.

#### 8.2.2 Definition of keyword-level sampling strata

Although we did a frequency-based analysis of keyword-wise data distribution in Section 4.8 we need to define sampling strata based on noise behaviour not just frequency. Hence, we divided the set of 19 keywords into 4 groups as defined below (with reference to serial numbers mentioned in Table 12).

1. Main keyword: *Recommend* (Sr. 1)
2. Synonyms: 14 Keywords (Sr. 2 −15)
3. Antonyms: 4 Keywords (Sr. 16–19)
4. Special-interest keyword: *Champion* (for gaming related variable names in code snippets) (Sr. 12) and *support* (for supported features of any software or error/bug resolution) (Sr. 9).

Using the total count of each keyword appearing in SORD (taken from Table 12), we selected a subset of keywords for sampling including the seed keyword (recommend), 4 synonyms (suggest, suitable, favor, advocate), 2 antonyms (oppose, condemn), 2 special interest keywords (support, champion), respectively. The results are shown in Table 17.

**Table 17:** Sample Keyword Selection.

Sr.	Keyword	Keyword Group	Included in Sampling	Total Occurrences	Frequency Range
5	Suggest	Synonym	✓	4079,677	High frequency
9	Support	Special interest	✓	3828,689	
14	Commend	Synonym		1542,826	
1	Recommend	Recommend	✓	1534,511	
18	Reject	Antonym		313,794	Mid-frequency
2	Advise	Synonym		266,591	
6	Suitable	Synonym	✓	232,832	
15	Propose	Synonym		200,559	
8	Favor	Synonym	✓	174,480	
16	Oppose	Antonym	✓	156,076	
10	Urge	Synonym		124,988	
4	Adopt	Synonym		77,191	
11	Promote	Synonym		48,582	Low frequency
3	Advocate	Synonym	✓	12,175	
13	Endorse	Synonym		11,275	
12	Champion	Special interest	✓	10,063	
7	Praise	Synonym		3849	
19	Disapprove	Antonym		1509	
17	Condemn	Antonym	✓	953	

### 8.3 Sample size determination

To quantitatively analyse the prevalence of false positives in the extracted recommendations related questions, answers and comments a manual annotation was performed on a subset of the dataset. Given the large scale and heterogenous nature of the data i.e. spanning multiple files (QuestionsTitle, QuestionsBody, Answers and Comments) and extraction methods (exact and substring matching), a total sample size of 800 records was selected as a practical yet statistically meaningful annotation budget. This sample size balances feasibility of manual inspection with sufficient coverage to estimate false positive rates across diverse keyword categories and data sources (questions, answers and comments). The sampling design was further structured to ensure representation of high, mid and low frequency keywords as well as known noise inducing contexts such as variable naming and technical log artifacts.

### 8.4 Sample allocation strategy

#### 8.4.1 Sample allocation across dataset files

To estimate false positive rates in the keyword-based dataset, we employed a hierarchical stratified sampling strategy that ensures representation across dataset files, keyword groups, and individual keywords. We first defined a total sample size of N=800, which was distributed across the eight dataset files according to their relative sizes in terms of number of records (given in Table 7).

Let $N_{f}$ denote the number of records in file f, and $N_{\text{total}}$ the total number of records across all files. The initial file-level allocation was computed as:

$$n_{f}=800\cdot \frac{N_{f}}{N_{\text{total}}}$$

However, purely proportional allocation resulted in very small sample sizes for certain strata (particularly QuestionsTitle_Contain and QuestionsTitle_LikeMinusContain), which may lead to unreliable estimation. To avoid under-representation of small data sources, a minimum constraint of 30 samples per file was enforced. After applying this constraint, the remaining samples were redistributed proportionally across larger files (QuestionsBody, Answers, and Comments files) to maintain the total sample size of 800. The final allocation across dataset files is given in Table 18.

**Table 18:** Sample Distribution Across Files.

File	No. of Records	Sample Size	Sample Size with Minimum Sample Constraint
QuestionsTitle_Contain	73,901	∼6	30
QuestionsTitle_LikeMinusContain	90,685	∼7	30
QuestionsBody_Contain	1135,148	∼87	85
QuestionsBody_LikeMinusContain	1511,800	∼115	110
Answers_Contain	2228,118	∼170	160
Answers_LikeMinusContain	1427,770	∼109	105
Comments_Contain	1900,242	∼145	135
Comments_LikeMinusContain	2114,468	∼161	145
**Total**	**10,482,132**	**800**	**800**

#### 8.4.2 Sample allocation across selected keywords

Instead of sampling across all extracted keywords, a representative subset of nine keywords was selected to capture semantic diversity and known noise sources. This includes one main keyword, four positively related keywords, two negatively related keywords, one gaming-related keyword, and one technical log-related keyword. The total sample size (N=800) was allocated across these selected keywords proportionally to their frequency in the dataset. Let $N_{k}$ denote the total occurrences of keyword k, and $\sum_{k}N_{k}$ the total occurrences across all selected keywords. The number of samples assigned to keyword kis given by:

$$n_{k}=800\cdot \frac{N_{k}}{\sum_{k}N_{k}}$$

Within each keyword, samples were further distributed across dataset files proportionally to their occurrence in each file, and records were sampled from the textual field using substring matching. To ensure adequate representation of low-frequency keywords in the annotated subset, a minimum sampling threshold was applied during keyword-level allocation. Specifically, each selected keyword was assigned at least 10 samples, regardless of its relative frequency in the dataset. For keywords whose proportional allocation fell below this threshold, the sample size was increased to meet the minimum requirement. The excess samples introduced through this adjustment were compensated by proportionally reducing the allocation of high-frequency keywords, thereby preserving the overall sample size of 800 while maintaining representation across all selected keywords. The corresponding values for $n_{k}$ are given in Table 19.

**Table 19:** Sample Distribution Across Keywords.

Sr.	Keyword	Total Occurrences ($N_{k})$	Samples Size (Raw) ($n_{k})$	Sample Size with Minimum Sample Constraint
5	Suggest	4079,677	325.42	290
9	Support	3828,689	305.4	270
1	Recommend	1534,511	122.4	110
6	Suitable	232,832	18.57	25
8	Favor	174,480	13.92	20
16	Oppose	156,076	12.45	15
3	Advocate	12,175	0.97	10
12	Champion	10,063	0.8	10
17	Condemn	953	0.08	10
	**Total (**$\sum_{k}N_{k})$	**10,029,456**	**800**	**760**

#### 8.4.3 Sample allocation across selected keywords and dataset files

Following keyword-level allocation, samples for each keyword were distributed across dataset files proportionally to their frequency within each file. Specifically, for a given keyword, the number of samples assigned to each file was computed based on the relative occurrence of that keyword in the file compared to its total occurrences across all files using the following equation.

For each keyword kand file f:

$$n_{k,f}=n_{k}\cdot \frac{N_{k,f}}{N_{k}}$$

$n_{k}$: total samples assigned to keyword k(e.g., 290, 270, etc.)

$N_{k,f}$: number of records in file fcontaining keyword k (already given in Table 12)

$N_{k}$: total records containing keyword kacross all files

The resulting values were rounded to integers, and minor adjustments were applied to ensure that the total number of samples per keyword remained consistent. Records were then randomly sampled from each file by selecting entries in which the keyword appeared within the textual field.

Although the target sample size for manual annotation was set to 800 records, the final retrieved dataset consisted of 760 samples (see Table 20). This difference arises naturally from the keyword-based sampling process over free-text data. Since sample selection depended on the presence of specific keywords within textual fields across multiple dataset files, some of the planned (keyword, file) allocations could not be fully satisfied due to insufficient available occurrences in certain cases. Low-frequency keywords and certain file combinations did not contain enough additional eligible records beyond those already assigned through proportional allocation. As a result, the final sample size reflects the actual availability of keyword occurrences in the dataset while still preserving the intended sampling structure across keywords and files.

**Table 20:** Sample Distribution of Selected Keywords Across Dataset Files.

File	Recommend	Suggest	Suitable	Favor	Advocate	Oppose	Condemn	Champion	Support	Row Total
QuestionsTitle_Contain	0	0	1	0	0	0	0	0	4	**5**
QuestionsTitle_LikeMinusContain	1	1	0	0	0	0	0	0	3	**5**
QuestionsBody_Contain	5	23	6	1	1	0	1	1	44	**82**
QuestionsBody_LikeMinusContain	10	55	0	4	1	3	2	3	29	**107**
Answers_Contain	40	46	9	3	3	1	1	1	68	**172**
Answers_LikeMinusContain	15	34	0	5	1	5	2	2	37	**101**
Comments_Contain	25	46	8	3	3	0	2	1	51	**139**
Comments_LikeMinusContain	14	85	1	4	1	6	2	2	34	**149**
**Column Total**	**110**	**290**	**25**	**20**	**10**	**15**	**10**	**10**	**270**	760/800

### 8.5 Annotation-based error analysis and statistical estimation

Each sampled record was manually annotated by a human labeller as either true positive (seeking or providing any sort of recommendation) or false positive (keyword triggered by non-recommendation context such as variable names, logging statements, or technical artifacts). Out of 760 selected samples, 454 were true positives and 306 were false positives. The following formulas were used to calculate the false positive rate (FPR) and precision.

$$False\;Positive\;Rate\;\left(FPR\;\%\right)=\frac{FP}{TP+FP}\;\times \;100=\;\frac{306}{760}\times \;100=40.26\%$$

$$Precision\left(\%\right)=\;\frac{TP}{TP+FP}\times \;100=\;\frac{454}{760}\;\times \;100=59.74\%$$

For a 95% confidence interval, the margin of error is:

$$\text{margin}\approx 1.96\;\times \sqrt{\frac{p\left(1-p\right)}{n}}=\;1.96\;\times \sqrt{\frac{0.403\left(1-0.403\right)}{760}}=\;0.03487\;≅0.035\;\left(3.5\%\right)$$

Where:

• **p** = fraction of false positives (e.g., 0.403)
• **n** = sample size

The overall precision across all sampled instances is 59.7% (95% CI: 56.2–63.2%), with a false positive rate of 40.3%. However, false positives were not uniformly distributed across keywords. Hence, the resultant aggregate is heavily influenced by a single keyword (support), which accounts for approximately 87% of all false positives. While most keywords exhibited low false positive rates (e.g., recommend: 2.73%, suggest: 3.10%), certain keywords showed substantially higher error rates (e.g., suitable: 24%, favor: 50.0%, champion: 80.0%) (presented in Table 21). This suggests that ambiguity in specific keywords—particularly those frequently used in code variables or system-generated logs—contributes disproportionately to noise in the dataset (Table 22).

**Table 21:** Keyword-wise Error Estimation.

Keyword	True Positives	False Positive	Total	FPR (%)	Precision (%)
Recommend	107	3	110	2.73	97.27
Suggest	281	9	290	3.1	96.9
Suitable	19	6	25	24	76
Favor	10	10	20	50	50
Advocate	9	1	10	10	90
Oppose	13	2	15	13.33	86.67
Condemn	9	1	10	10	90
Champion	2	8	10	80	20
Support	4	266	270	98.52	1.48

**Table 22:** File-wise Error Estimation.

File Name	Including Support Keyword					Excluding Support Keyword				
	TP	FP	Total	FPR(%)	Precision(%)	TP	FP	Total	FPR(%)	Precision(%)
QuestionsTitle_Contain	1	4	5	80	20	1	0	1	0	100
QuestionsTitle_LikeMinusContain	1	4	5	80	20	1	1	2	50	50
QuestionsBody_Contain	34	48	82	58.54	41.46	34	4	38	10.53	89.47
QuestionsBody_LikeMinusContain	68	39	107	36.45	63.55	68	10	78	12.82	87.18
Answers_Contain	97	75	172	43.6	56.4	97	7	104	6.73	93.27
Answers_LikeMinusContain	55	46	101	45.54	54.46	55	9	64	14.06	85.94
Comments_Contain	86	53	139	38.13	61.87	84	4	88	4.55	95.45
Comments_LikeMinusContain	112	37	149	24.83	75.17	110	5	115	4.35	95.65

Excluding the *support* keyword, the remaining keywords exhibit substantially higher precision, indicating that false positives are concentrated in specific ambiguous terms (e.g., variable names or logging artifacts). Removing the 266 samples corresponding to the support keyword, the results for precision and false positive rate changed significantly from 40.26% to 8.16% and 59.74% to 91.84% respectively with a 95% confidence interval of 2.42% which was previously 3.5% (as calculated below).

$$False\;Positive\;Rate\;\left(FPR\;\%\right)=\frac{FP}{TP+FP}\;\times \;100=\;\frac{40}{490}\times \;100=8.16\%$$

$$Precision\left(\%\right)=\;\frac{TP}{TP+FP}\times \;100=\;\frac{450}{490}\;\times \;100=91.84\%$$

For a 95% confidence interval, the margin of error is:

$$\text{margin}\approx 1.96\;\times \sqrt{\frac{p\left(1-p\right)}{n}}=\;1.96\;\times \sqrt{\frac{0.0816\left(1-0.0816\right)}{490}}=\;0.024\;\left(2.42\%\right)$$

Table 22 presents false positive rate and precision results for each of the 8 files included in SORD. The precision significantly improved when we excluded the support keyword from the sample data corresponding to each file. As the per-file and the per-keyword analysis reveals substantial variation in precision with most keywords achieving high precision (e.g., recommend and suggest above 96%), and a small subset of keywords exhibiting significantly lower precision, it suggests that false positives are not uniformly distributed but are instead concentrated in a few ambiguous keywords, likely due to their frequent occurrence in variable names, system logs, or non-recommendation contexts.

Additionally, certain terms exhibited domain-specific usage patterns that were not anticipated during the initial keyword selection process. These patterns emerged only during manual annotation, where contextual inspection revealed that some keyword occurrences were semantically valid within specialized technical contexts, further contributing to observed variability in precision across keywords. For instance, we expected the term support to appear in the context ‘I support xyz’s opinion/suggestion regarding abc’. However, it occurred in the context of missing or existing feature support, thanking for support, contacting customer support (of the product under discussion), as suggested by the support team, universally supported, supported version, as part of class, variable, package and exception names and unsupported formats to name a few. These findings highlight that, although the overall dataset maintains high precision, keyword selection plays a critical role in determining data quality. It further supports the effectiveness of keyword-based filtering for most terms, while highlighting the need for additional disambiguation for specific keywords. Hence, future refinements may focus on disambiguating high-noise keywords or applying additional filtering strategies to reduce false positives.

## 9 Threats to validity

### 9.1 Construct validity

Construct validity threats involve discrepancies between the theoretical definitions and practical observations which have been mitigated by considering definitions of various recommendation related keywords (as described in Table 5). A keyword-based filtering has been done to identify potential posts and comments containing developer preferences and sample records were analyzed to evaluate the possible forms of false positives and irrelevant results.

### 9.2 External validity

External validity results pertain to the generalizability of findings. Being the oldest and most popular Q&A site of the Stack Exchange Network, we restricted our scope to Stack Overflow. However, the Stack Exchange Network (which hosts Stack Overflow) currently hosts 182 Q&A sites out of which 77 are technology related. Excluding Stack Overflow (which has been considered in this paper), its Russian, Japanese, Spanish and Portuguese variants and the Meta Stack Exchange site which deals with meta-discussions related to Stack Exchange based Q&A sites, the same approach can be applied on the data dumps of 71 other technology related Stack Exchange based Q&A sites, as all these sites follow a uniform database schema [20]. Although there can be other technology related Q&A sites that are not part of the Stack Exchange Network, our chosen sample provides a good representation of the overall population (of Q&A sites) as the Stack Overflow data dump [1] considered for curation of SORD contained data from 2008 to 2025, making SORD one of its kind, large-scale and most extensive software recommendation focused dataset. Hence, Stack Overflow (being the oldest and the most popular Q&A site among developers) can be considered a rich source of information regarding developer preferences and can be considered a representative sample of software development community. Therefore, the findings can be easily generalized.

### 9.3 Internal validity

Internal validity threats pertain to internal factors affecting our results. We applied keywords-based filtering on Stack Overflow posts (questions and answers) and comments by applying MS SQL Server’s full text search and wildcard search (as shown in Box 14, Box 15 – Box 16) without applying any text preprocessing. Code snippets found in question body and answers were not removed as we were not expecting any of the recommendation related keywords (mentioned in Table 5) to be found in code snippets (used by developers as variable names). Similarly, user mentions were not removed from comments. Resultantly, some false positives were included in the filtered records e.g. results containing *urge* as a substring such as '*@Pierre-OlivierBourgeois So should I move my question*?' and those containing *champion* as a variable name in questions with code snippets regarding some game development related questions.

## Limitations

• The data curation process involves the use of SQL Server as the database management system. However, SQL Server Express Edition (free to use) is limited by 10GB database size [29]. Hence, we used the SQL Server Developer Edition to curate the dataset. However, it can’t be used in a production environment.
• The dataset currently filtered the Questions (title, body), Answers and Comments based on the chosen set of keywords without applying any text preprocessing on the relevant fields. Questions (particularly body), Answers and even some Comments may contain code snippets, and the keywords may be present in those code blocks as well. This limitation can be overcome by removing code blocks to refine the results.
• While curating additional metadata from the Stack Overflow data dump associated with SORD, we extracted the set of unique tags associated with recommendations related data (present in \additional-metadata\FilteredTags.csv). In FilteredTags, we observed the presence of several closely related tags (and there will be even more such tags in Stack Overflow data dump) e.g. .net-1.0, .net-1.1, .net-4.5.2, .net-9.0 and so on. However, in their current form, they exist as independent entities and their implicit/explicit mutual relationship is not readily available for utilization. As SORD is curated from the Stack Overflow data dump, the absence of a tag-hierarchy in the Stack Overflow tagging system is also reflected in the flat structure of FilteredTags. The absence of such hierarchy may hinder the effectiveness of potential recommendation models built using this data. In its current form, topics related to python, python-3.x and python-2.7 are treated as distinct tags, resulting in fragmented content which may lead to less accurate recommendations. This limitation can lead to content being missed or grouped incorrectly. For instance, a user searching for information on ``Python'' may miss relevant answers tagged with ```Python 2.7” or ``Python 3.x,'' reducing the effectiveness of the model. Recommendation models typically rely on content similarity, user preferences, and tagged keywords to suggest relevant content. If the model cannot recognize the relationship between ``Python,'' ``Python 2.7,'' and ``Python 3.x'' as related topics, it may suggest irrelevant or less useful content. For example, if a user asks about ``Python 2.7” and the recommendation system has no way of linking it to ``Python,'' it may miss content related to general ``Python'' programming that could still be highly relevant to the user’s question. Without tag-hierarchy or linking of morphologically variant tags, the clustering of similar content is hindered. This could lead to less accurate content groupings, where related answers, even though relevant to the same information need, are spread across different clusters because they are tagged with slightly different terms. Consequently, the recommendation model will struggle to suggest the most relevant answers, as it lacks a holistic view of the topic. Future work could address this by implementing tag normalization or hierarchical relationships, improving the grouping and relevance of recommendations based on the content.
• The data curation pipeline involves two main stages: converting the XML based Stack Overflow data dump to MS SQL Server and subsequent data filtering and keywords-based extraction using SQL Server. A significant portion of the overall computational cost is incurred during the data dump processing stage, which is inherently dependent on the size and content distribution of the specific dump version used. As different Stack Overflow dump releases may vary in scale and in the availability of relevant recommendation-related content, the total processing time is not fixed across versions. In contrast, the filtering and extraction stage is relatively lightweight and contributes minimally to overall runtime. Due to this inherent variability across dataset versions and hardware configurations, standardized runtime benchmarking across different systems is not particularly meaningful for this pipeline. However, we note that the computational requirements remain manageable and primarily scale with the size of the input dump [2].

## Future work

Our work can be extended in several diverse directions. Firstly, the questions containing one or more of the recommendations related keywords in question title, body or both with an accepted answer or at least some positively scored answers can be utilized to create a benchmark data set for the retrieval evaluation of potential RSSE. This set of questions can be further augmented by integrating questions (and their accepted answers) from the Software Recommendations site, particularly created to ask and answer software recommendations related questions.

Second, asking for any sort of recommendations related questions on Stack Overflow is considered off-topic and any such questions get closed as they often tend to attract opinionated and subjective answers [3]. However, out of 73,901 questions containing one or more recommendation related keywords in question title filtered through exact matching i.e. QuestionsTitle_Contain, only 3973 questions were closed. Hence, questions present in SORD can be used to train Stack Exchange bots to more accurately identify recommendation related questions and automatically migrate such questions to Software Recommendations site instead of closing them or letting the questioners get stuck in the cumbersome question migration policies as discussed in a recent study [18]. This will not only help questioners get their recommendation related questions answered without compromising on the healthy knowledge sharing and moderation policies of Stack Overflow but will eventually help attract knowledgeable Stack Overflow users to the Software Recommendations site, improving its percent answered rate. It is worth noting that the Software Recommendations site has been the least answered site of Stack Exchange Network for the last 5 years with currently 57% answered rate.

Third, although the analysis of sample comments in Section 7 extracted through a rather crude keywords-based approach demonstrated promising results, it is still prone to error because we have not performed any kind of text preprocessing step before curating SORD from the Stack Overflow data dump. Hence, future versions of SORD will focus on removing code snippets potentially present in questions’ body and answers. Furthermore, to improve rigor, we intend to improve the data extraction method by incorporating advanced text processing techniques to better contextualize relevance rather than exact keyword presence.

Fourth, the framework can be expanded to support dynamic keyword management allowing automatic extraction and ranking of important terms instead of relying on a predefined set of keywords. This would make the dataset more adaptive. Furthermore, we plan to analyze the sensitivity of the dataset construction process to keyword selection and its impact on data coverage and precision. We also aim to explore adaptive and machine learning-based filtering approaches to reduce reliance on manually defined keyword lists and improve robustness in identifying relevant recommendation-related content.

Fifth promising direction is to extend the analysis across multiple sources including the data dump of >70 other technology-based Q&A sites that are part of the Stack Exchange Network. Furthermore, by introducing weighting schemes different keywords based on different contexts could provide more meaningful insight by reflecting their relative importance. This would further help in inferring ratings from the implicit reviews present in developer discussions regarding different software, tools, technologies, frameworks, libraries and so on.

Six, integrating named entity recognition, automatic aspect identification, opinion mining and other such techniques to filtering mechanisms will enhance the practical value of SORD. Furthermore, identification of obsolete content and version specific information included in SORD will further help in reducing noise. In this regard, linking variants of specific tags e.g. linking python-2.7 and python-3.x to python will help to categorize all python related questions, answers and comments present in SORD, which currently exist as related to three different entities i.e. python-2.7, python-3.x and python respectively. As programming languages evolve, older versions of a language (like Python 2.7) may become less commonly discussed, but the content related to these older versions remains valuable for historical context or for users maintaining legacy systems. If such content is tagged with an outdated or obsolete version-specific tag (e.g., ``python-2.7”), and that tag isn't linked to a more general ``Python'' tag, valuable information may be overlooked by recommendation systems or searches. By linking variants like ``Python 2.7” to a broader ``Python'' tag, we could ensure that this data remains discoverable, preserving its usefulness even as it becomes less prominent in current discussions.

Furthermore, introducing a tag-hierarchy will enable the community to develop multi-criteria recommender systems based on this data. With a tag-hierarchy in place, the recommendation model could not only recommend content based on a coarse-grained, high-level view (e.g., general ``Python'' content) but also allow for more fine-grained comparisons between specific versions (e.g., ``Python 2.7” vs ``Python 3.x''). If a user is looking for information specifically about the differences between versions (e.g., "What are the key differences between Python 2.7 and Python 3.x?"), a multi-criteria recommender could prioritize content that compares these versions in a detailed manner. On the other hand, if a user is simply interested in general Python content, the model could aggregate information from all versions (e.g., "Python," "Python 3.x," "Python 2.7") and present a broader, more holistic overview. The hierarchical structure would make it easier to tailor the recommendation process to the user's specific needs, whether they require detailed comparisons or a general understanding of the topic.

Seven, incorporating categorical information of entities discussed in questions, answers and comments included in SORD can help in realization of RSSE to help developers in their design choices at various stages of software development lifecycle (SDLC) e.g. choosing the right programming language and its frameworks/packages/libraries for achieving a particular goal.

Eight, we intend to do a systematic analysis of noise introduced by keyword-based filtering, including identifying common error types and their impact on dataset quality. We also plan to explore methods to mitigate such noise and improve the precision of extracted recommendation-related content.

Finally, we plan to extend the utility of the dataset by developing and evaluating downstream machine learning models, such as recommender systems and sentiment analysis frameworks. These efforts will enable a more comprehensive assessment of the dataset’s effectiveness in real-world applications and help identify opportunities for improving data quality and coverage. Additionally, we aim to explore advanced filtering approaches, including semantic and machine learning-based methods, to mitigate potential biases introduced by the current keyword-based filtering strategy.

## Ethics Statement

As the dataset was curated from Stack Overflow data dump which is available under Creative Commons CC BY-SA license (various versions, including 2.5, 3.0, and 4.0), our work is aligned with the ethical requirements for publication in Data in Brief.

## CRediT Author Statement

**Arjumand Fatima:** Conceptualization, Methodology, Software, Validation, Investigation, Data Curation, Writing – Original Draft; **Onaiza Maqbool:** Supervision, Writing- Reviewing and Editing.

## Data Availability

FigshareStack Overflow Recommendations Dataset (SORD) - Version 1.0 based on Stack Overflow Data Dump - October 2025 (Original data).
